# Novel dimeric dual-modality FAP-targeted agents with favorable tumor retention for image-guided surgery: a preclinical study

**DOI:** 10.1007/s00259-025-07626-z

**Published:** 2025-11-19

**Authors:** Giacomo Gariglio, Thomas Hasenöhrl, Katerina Bendova, Zbyněk Nový, Christine Rangger, Kai Kummer, Bradley D. Smith, Barbara Matuszczak, Milos Petrik, Clemens Decristoforo

**Affiliations:** 1 Department of Nuclear Medicine, Medical University of Innsbruck, 6020 Innsbruck, Austria; 2 Institute of Pharmacy, Department of Pharmaceutical Chemistry, University of Innsbruck, 6020 Innsbruck, Austria; 3 Institute of Molecular and Translational Medicine, Faculty of Medicine and Dentistry, Palacky University, 77900 Olomouc, Czech Republic; 4 Czech Advanced Technology and Research Institute, Palacký University, 77900 Olomouc, Czech Republic; 5 University Hospital Olomouc, 77900 Olomouc, Czech Republic; 6 Institute of Physiology, Medical University of Innsbruck, 6020, Innsbruck, Austria; 7 Department of Chemistry and Biochemistry, University of Notre Dame, 46556 Notre Dame, Indiana, USA

**Keywords:** FAP, PET, Fluorescence-guided surgery, Dual-modality imaging agent, Tumor retention

## Abstract

**Purpose:**

Complete and minimally invasive cancer surgery remains challenging. Targeting the fibroblast activation protein (FAP) offers valuable opportunities for surgical planning, intraoperative guidance and improved resection outcomes. Herein, we developed the first dimeric, dual-modality FAP-targeted imaging agents and investigated the influence of different near-infrared cyanine-7 dyes on their final properties.

**Methods:**

Four dual-modality ligands based on the Fusarinine C scaffold were synthesized. Their FAP specificity and retention were evaluated in cellular and xenograft tumor models. The most promising candidates were labelled with ^67/68^Ga and assessed *in vivo* at early time points by PET/CT imaging and by comparative SPECT/CT and NIR fluorescence imaging (FI) up to two days post-injection.

**Results:**

Distinct fluorophore influences on the properties of the final compounds were identified. The introduction of the s775z dye demonstrated a beneficial effect on the cellular uptake and on the *in vivo* biodistribution profile as revealed by the greatest improvement in blood clearance and the least off-target accumulation in liver and kidneys when compared to the control and to the other candidates respectively. *Ex vivo* experiments and *in vivo* PET/CT, SPECT/CT and FI studies in xenografted mice confirmed these findings and demonstrated sustained tumor uptake (> 7% ID/g and > 5% ID/g at 1 h and 1 day p.i. respectively) for ^67^Ga-s775z-FFAPi and ^67^Ga-IRDye-FFAPi.

**Conclusions:**

In this study we introduced and evaluated novel dimeric FAP-targeting agents for dual-modality applications. In the preclinical setting, within the group of compounds investigated, two candidates enabled tumor visualization through PET, SPECT and optical imaging, providing satisfactory background contrast after a single administration and supporting their potential for preoperative nuclear imaging and subsequent fluorescence-guided surgery.

**Supplementary Information:**

The online version contains supplementary material available at 10.1007/s00259-025-07626-z.

## Introduction

Surgical intervention continues to be central to cancer treatment, particularly for head and neck squamous cell carcinomas (HNSCCs) [1]. However, complete removal remains challenging due to ill-defined tumor margins and proximity to critical structures, leading to positive surgical margins (PSM) and increased recurrence risk. The National Comprehensive Cancer Network Guidelines 2024 emphasize the importance of achieving clear surgical margins in head and neck cancer (HNC) [2].

To support complete and minimally invasive resection, while preserving patient function and aesthetics, novel surgical guidance approaches are needed [3].

Preoperative positron emission tomography (PET) scans are routinely used in HNSCC patients to assess tumor extent, lymph nodes involvement, and metastases, providing a roadmap for the successful execution of the surgery [4]. Intraoperatively, near-infrared (NIR) fluorescence imaging (650–900 nm) allows real-time visualization of tumor boundaries, aiding surgical decision-making. Fluorescent probes targeting upregulated biomarkers in HNSCC are currently under clinical trial evaluation, including cetuximab-IRDye800CW and panitumumab-IRDye800CW [5–7].

Because nuclear and fluorescence imaging have complementary strengths, limitations and applications, their synergistic combination in a targeted dual-modality imaging agent holds promise for enabling complete and minimally invasive resection [8, 9]. Ideally such a probe, with a single biodistribution profile, provides consistent pre- and intraoperative information, enabling improved tumor localization and resection.

The fibroblast activation protein (FAP), a type II transmembrane serine protease, highly expressed in cancer-associated fibroblasts (CAFs) in more than 90% of epithelial tumors, has emerged as a valuable pan-cancer imaging target [10]. Following Haberkorn’s pioneering work, several quinoline-based fibroblast activation protein inhibitor (FAPI) radiotracers have been recently developed and showed diagnostic success in various tumors, including the HNCs, with advantages over the “gold standard” [^18^F]FDG [11–13].

The near-universal infiltration of CAFs into solid tumors makes FAP a promising target for surgical guidance techniques as well. In particular, HNSCC is characterized by a dense fibrotic stroma rich in CAFs. This fibrotic tissue obscures the tumor's true extent, as individual cancer cells often invade the stroma and form microscopic buds at the invasive front [13–16]. Consequently, a surgical guidance technique based on FAP expression in the peritumoral stroma, rather than on a cancer cell-specific marker, may better consider the full extent of the tumor, minimizing postoperative malignant residues. Therefore, FAPI radiotracers have become promising scaffolds for developing targeted probes suitable for fluorescence-guided surgery of HNCs and other indications [17–20]. Dual modality probes have been developed for different targets and recently also first examples of dual-modality imaging probes targeting FAP have also been reported [21–25].

An ideal dual-modality imaging agent should be administered in a single injection, rapidly accumulate in tumor lesions, and persist long enough to enable both preoperative nuclear imaging and subsequent image-guided surgery (e.g., one day post-injection). Compared to their monomeric counterparts, FAPI-based homodimers, like DOTAGA.(SA.FAPi)_2_ and BiOncoFAP, offer prolonged tumor retention while preserving elevated tumor-to-background ratios [26, 27]. Therefore, they are attractive platforms for the development of bimodal probes.

Both the introduction of a fluorophore and subtle modifications in its chemical structure can markedly influence the pharmacokinetic profile of a targeting molecule [28–31]. In the case of hybrid ligands, this phenomenon has been investigated in detail for candidates targeting PSMA and αvβ3 integrin, overall highlighting the importance of careful fluorophore evaluation for targets where such studies are still lacking [32–34].

In this project, we developed the first dimeric PET/FI dual-modality imaging agents targeting FAP. We incorporated four NIR fluorophores with distinct structural features into the same Fusarinine C (FSC)-based scaffold and investigated their specific influences on both *in vitro* and *in* *vivo* properties. As the most representative examples of Cy7 fluorophores, we selected SulfoCy7 and the FDA-approved IRDye800CW, the latter of which carries a highly anionic surface charge due to the presence of four sulfonate groups. Additionally, we included s775z and ZW800 as two examples of zwitterionic Cy7 dyes. Notably, the s775z dye differs from ZW800 by offering improved chemical and photostability, along with additional shielding arms that minimize undesired molecular interactions [35, 36]. The resulting dual-modality ligands were characterized using FAP expressing cells and xenografted models to assess their potential for preoperative imaging and surgical guidance of FAP-positive lesions.

## Materials and methods

### Cell-based experiments

Cell lines and culturing conditions are described in the Supplementary Material.

### Cell uptake studies

The cell internalization of the Gallium-68 and Zirconium-89 radiolabelled compounds was measured on HT1080 and HT1080hFAP cells. 1.8 × 10^5^ cells per well were seeded in 24-well culture plates (TC-Platte 24, SARSTEDT AG & Co. KG, Nümbrecht, Germany) and grown for 48 h. On the day of the experiment, the cells in each well were washed twice with 400 µL of culturing medium and then incubated with the radioactive compound (1 nM/well final concentration) for 1 h at 37 °C. After the incubation, the medium was removed and the cells rinsed twice with 400 µL of PBS/0.5% (w/v) Bovine Serum Albumin (BSA). To retrieve the membrane-bound fraction, cells were washed twice with 400 µL of 50 mM glycine buffer (pH 2.8). Eventually, the cells were lyzed by adding two times 400 µL of 1 M NaOH to determine the internalized fraction of radioligand. All fractions were measured in the γ-counter and the percentage of internalized and membrane bound radiocompound in relation to the total radioactivity added to the cells was reported. The reported values were obtained from the results of three independent experiments.

### Fluorescence microscopy studies

Fluorescence imaging was performed with an Olympus IX83 inverted fluorescence microscope (Olympus America, Center Valley, PA, USA) equipped with a 20X plan-apochromat air objective. Fluorescent tracer uptake was analyzed on HT1080hFAP and HT1080 cells. 1.2 × 10^5^ cells per well were seeded in µ-Slide 8 well plates (Ibidi GmbH, Gräfelfing, Germany) 48 h prior the experiment. The cells were washed with fresh culturing medium and then incubated with metal-free dual-modality agent (500 nM/well final concentration) for 1 h at 37°C. After washing, HOECHST 3342 (Thermo Fisher Scientific, Vienna, Austria) was added 5 min before microscopy to a final concentration in each well of 20 µM. Images were captured using identical microscope settings (20X magnification, λexc = 395/405 nm for HOECHST and λexc = 730/740 nm for the fluorescent tracers) and uniformly processed with the open access software FIJI (ImageJ, Version 1.53c, National Institute of Health (NIH), Bethesda, MA, US) [37].

### Radioactive-based cell efflux studies

For cell efflux studies, 1.0 × 10^6^ HT1080hFAP cells were seeded in 6-well plates and cultivated for 48 h. On the day of the experiment, the cells in each well were washed twice with 1.5 mL of culturing medium and then incubated with the Gallium-68 labelled radiotracer at a final concentration of 1 nM/well for 1 h at 37 °C. The medium containing the unbound radiotracer was then replaced with either fresh medium or a 1 µM FAPI-46 solution in cell medium for blocking condition.

Subsequently, at specific time points, the radioactive medium was removed and collected together with two PBS/0.5% (w/v) BSA washes before adding 1 mL of fresh medium or FAPI-46 blocking solution. Finally, 2 h after the end of the first incubation, the cells were lyzed with 1 mL of 1 M NaOH. All collected fractions were measured in the γ-counter and the percentage of cell-associated activity at different time points relative to the total, corresponding to 1 h after incubation, was reported. The number of experimental replicates performed for each assay is specified in the respective figure legends.

### Fluorescence-based cell efflux studies

HT1080hFAP cells (1 × 10^6^ cells/well) were seeded in 6-well plates and cultured for 48 h. On the day of the experiment, cells were washed once with 1.0 mL of culturing medium. Afterwards they were incubated with the non-radioactive tracer (s775z-FFAPi or IRDye-FFAPi) at a final concentration of 20 nM/well for 1 h at 37 °C. After incubation, the medium was removed, and cells were washed once with 0.5 mL of PBS containing 0.5% (w/v) BSA. Fresh medium (1.0 mL) or blocking solution (medium supplemented with 1 µM FAPI-46) was then added, and cells were further incubated for various time intervals (0–5 h). At each time point, the medium was removed and collected together with a subsequent 0.5 mL of PBS/0.5% (w/v) BSA wash. Cells were lyzed with 1.5 mL of EtOH/H_2_O (1/1, v/v), and 150 µL aliquots of each collected fraction were transferred to black, flat-bottom 96-well plates for fluorescence measurement using a Tecan Spark multimode plate reader (Tecan, Männedorf, Switzerland; top reading; excitation wavelength 733 ± 10 nm, emission wavelength 802 ± 10 nm; 50% mirror; multiple reads per well, 3 × 3 circle pattern).

Fluorescence intensity values were background-corrected using the corresponding culturing medium or EtOH/H_2_O mixture. The percentage of cell-associated fluorescence at each time point was calculated relative to the total signal measured at 1 h post-incubation. Each experiment was performed in two technical replicate wells, each measured in triplicate.

### *Ex vivo* biodistribution experiments, small animal imaging studies

General information about animals, housing and handling are provided in the Supplementary Material.

For the *ex vivo* biodistribution study on healthy female BALB/c mice, 4 animals per group were injected with 0.10 nmol of Gallium-68 labelled tracer (0.5 MBq) and then sacrificed after 1 h. The animals were allocated to groups without randomization. The organs of interest were extracted, weighed, and measured in the γ-counter. Results were expressed as percentages of injected dose per gram tissue (% ID/g).

For the induction of tumor xenografts, 2 × 10^6^ of HT1080hFAP or HT1080 cells in 100 μL appropriate medium were subcutaneously injected in the right and left flank of each mouse (athymic female BALB/c nude), respectively. The tumors were allowed to grow until they had reached a volume of 0.3 to 0.8 cm^3^. To achieve comparable baseline tumor sizes between groups, mice were divided into strata based on tumor size and assigned to groups to balance the distribution of tumor sizes. To evaluate *ex vivo* biodistribution on tumor models, 3 mouse xenografts were injected with 0.25 nmol of Gallium-67 labelled tracer (0.4 MBq) and then sacrificed after 1 h, 4 h and 1 day. For the PET/CT imaging study one xenograft-bearing mouse (tumor volume in the range 0.6–1.0 cm^3^) was injected with 1.0 nmol of Gallium-68 labelled tracer (5–8 MBq). Static PET/CT images of the anesthetized animal in prone position were acquired with a Mediso nanoScan PET/CT small-animal imaging system (Mediso Medical Imaging Systems, Budapest, Hungary) at 1 h and 2 h p.i. Image reconstruction was performed via Mediso Tera-Tomo 3D PET iterative reconstruction (Mediso Medical Imaging Systems, Budapest, Hungary). The images were visualized, processed, and quantified in Mediso InterView FUSION (Mediso Medical Imaging Systems, Budapest, Hungary). The images were normalized to injected activity and animal weight. The results were expressed as percentages of injected dose per gram tissue (% ID/g). For the comparative imaging study (SPECT and *in vivo* fluorescence imaging), one xenograft-bearing mouse (tumor volume in the range 0.2–0.7 cm^3^) was injected with 1.5–2 nmol of Gallium-67 tracer (15–17 MBq) and imaged at various time points up to 2 days p.i. Static SPECT/CT images of the anesthetized mouse in prone position were acquired on Mediso nanoScan SPECT/CT small-animal imaging system (Mediso Medical Imaging Systems, Budapest, Hungary). Image reconstruction was performed via Mediso Tera-Tomo 3D normal dynamic range (Mediso Medical Imaging Systems, Budapest, Hungary). The images were visualized, processed, and quantified in Mediso InterView FUSION (Mediso Medical Imaging Systems, Budapest, Hungary). Near-infrared *in vivo* fluorescence imaging was conducted with an *in vivo* MS FX PRO small-animal imaging system (Bruker Biospin Corporation,Woodbridge, CT, USA) and image analysis was performed with Bruker MI SE software v. 7.1.1.20220 (Bruker Biospin Corporation,Woodbridge, CT, USA). The mice were imaged in the supine position at various time points using a filter set with excitation wavelength of 720 nm and an emission wavelength of 790 nm. Acquisition parameters were kept constant across all scans (exposure time = 5 s, f-stop = 2.8, field of view = 100 mm, binning = 4 × 4). The fluorescence emission was reported as photons/s/mm^2^.

## Results

### Synthesis of the labelling precursors

The siderophore FSC was isolated from *Aspergillus fumigatus* ∆sidG cultures, and an iron salt was subsequently added to form the corresponding metal complex [38]. This procedure is essential to prevent hydroxamate groups from participating in side-reactions during subsequent synthesis steps. The FAPI alkyne derivative was synthesized using a modified approach based on a previously reported procedure [39].

**Fig. 1:**
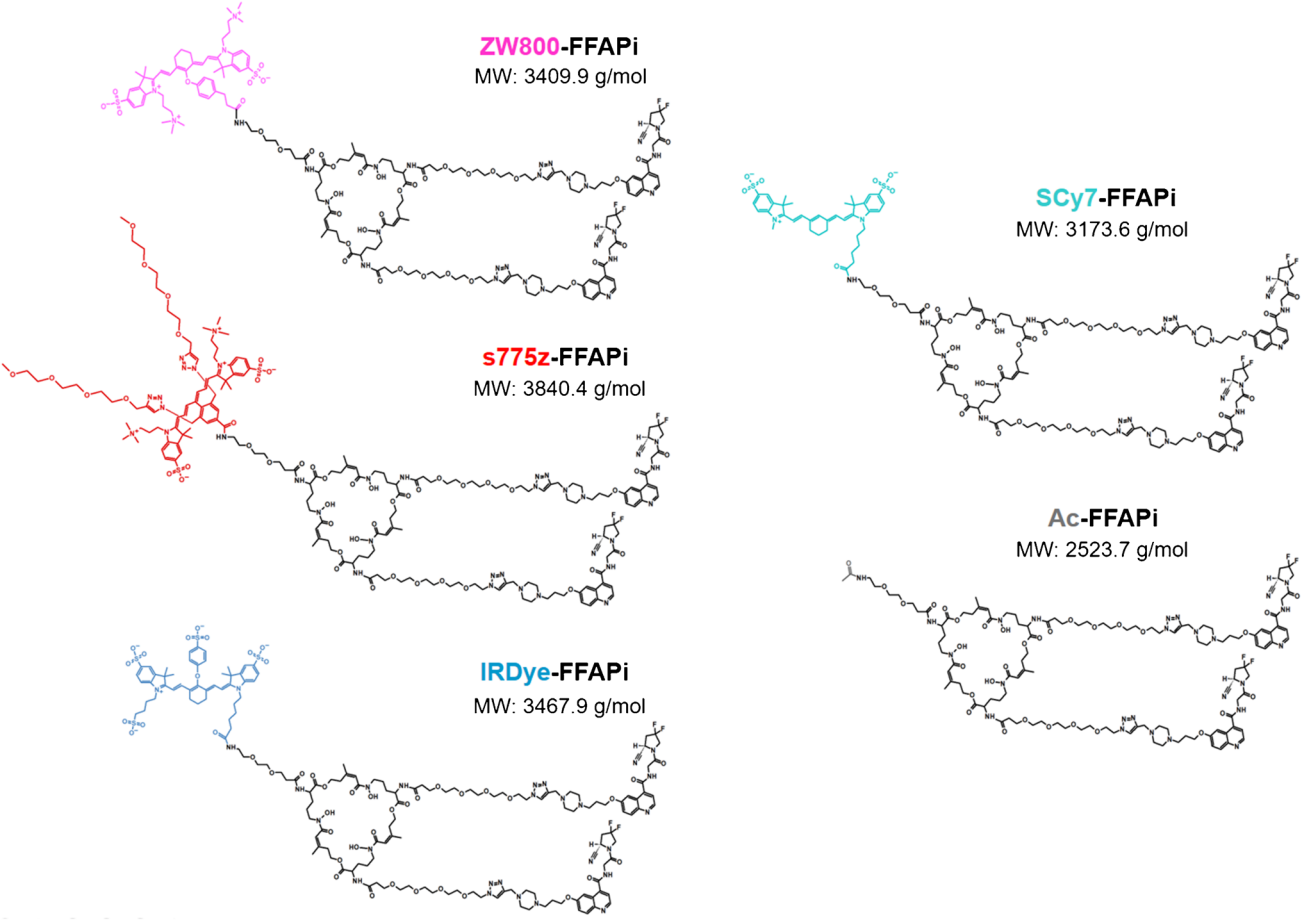
Chemical structures of the investigated compounds

The synthetic route of the labelling precursors is described in detail in the Supplementary Information and shown in Fig. S8. First the chelator’s three amine groups were derivatized via conventional amide coupling to introduce a PEG2-amine and two PEG4-azide linkers. Two units of FAPI alkyne derivative were subsequently introduced by Cu(I)-catalyzed azide–alkyne cycloaddition (CuAAC) click reaction. The resulting intermediate was then conjugated with the selected NIR fluorophores through amide coupling. Eventually, the coordinated metal was removed with an excess of EDTA to provide the corresponding labelling precursor in moderate yields (44–57%) and sufficient chemical purity (96–98%). Analogously, an acetylated ligand lacking the fluorophore, Ac-FFAPi, was prepared as control (60% yield and 98% purity). All ligands described in this study are summarized in Fig. 1. Their photophysical properties (absorption/emission maximum) were consistent with those of the original dyes (Table S5).

### Radiolabelling

All precursors were radiolabelled with Gallium-68 at RT within 10 min in high radiochemical yields (RCY > 99.5%, Fig. S28) and radiochemical purities (RCP > 94%, Fig. S27) with the exception of IRDye-FFAPi and SCy7-FFAPi (RCP: 90.7 and 72.8% respectively) for which radiolysis side-products were observed. Quantitative labelling with Gallium-67 (RCY: > 99%, Fig. S30) was achieved by heating at 80 °C for 10 min, whereas Zirconium-89 (RCY: > 99%; RCP: 98.4%; Fig. S29) required heating at 40 °C for 30 min. Due to the high labelling efficiencies achieved with these radionuclides no additional purification was performed and the radiolabelled tracers were directly used in all experiments.

### *In vitro* characterization

Table 1 shows the results of the lipophilicity, protein binding and stability in human serum determination. All investigated Gallium-68 labelled compounds exhibited high stability in human serum, with less than 4% radionuclide release up to 4 h p.i. Overall, comparable protein binding in the range 25–33% was found for all dual-modality candidates. Lower affinity for proteins was instead observed for the acetylated control (13–18%). As shown in Fig. S31, the LogD_pH7.4_ values of the ligands labelled with Gallium-68 ranged from −2.09 to −2.92. As expected, [^68^Ga]Ga-SCy7-FFAPi exhibited the lowest hydrophilicity. Comparable LogD_pH7.4_ values were determined for [^68^Ga]Ga-ZW800-FFAPi, [^68^Ga]Ga-IRDye-FFAPi and the [^68^Ga]Ga-Ac-FFAPi control (respectively, − 2.35 ± 0.07, − 2.45 ± 0.08 and − 2.51 ± 0.01). [^68^Ga]Ga-s775z-FFAPi exhibited the highest hydrophilicity in the group (− 2.92 ± 0.04), which was also notably and statistically higher than that of the control; thereby demonstrating the beneficial contribution of the fluorophore to this property. No significant difference in stability or binding to human proteins was observed for s775z-FFAPi when labelled with Gallium-68 or Zirconium-89 (Table 1). Interestingly, the Zirconium-89 labelled version exhibited lower hydrophilicity (− 2.01 ± 0.08) compared to its Gallium-68 counterpart. This result was unexpected, as the Zirconium (IV) complex of FSC carries a single positive charge, whereas the corresponding Gallium (III) complex is neutral. The underlying reason for this discrepancy remains unclear.

**Table 1:** Results of lipophilicity (LogD_pH7.4_), protein binding, and stability determination in human serum (% of intact radiotracer) for Gallium-68 labelled probes and for [^89^Zr]Zr-s775z-FFAPi

		[^68^Ga]Ga-Ac-FFAPi	[^68^Ga]Ga-SCy7-FFAPi	[^68^Ga]Ga-IRDye-FFAPi	[^68^Ga]Ga-ZW800-FFAPi	[^68^Ga]Ga-s775z-FFAPi	[^89^Zr]Zr-s775z-FFAPi
Lipophilicity	LogD_pH7.4_ ± SD	− 2.51 ± 0.01	− 2.09 ± 0.15	− 2.45 ± 0.08	− 2.35 ± 0.07	− 2.92 ± 0.04	− 2.01 ± 0.08
Protein binding (% ± SD)	1 h	13.3 ± 1.3	25.2 ± 3.9	30.6 ± 1.1	25.6 ± 0.6	30.6 ± 0.2	27.9 ± 1.9
	2 h	18.3 ± 2.1	26.5 ± 2.5	33.2 ± 3.1	26.1 ± 1.7	29.9 ± 0.8	25.0 ± 2.5
	4 h	17.2 ± 1.2	27.2 ± 2.7	30.9 ± 1.1	25.6 ± 1.9	30.0 ± 0.1	28.3 ± 2.2
Stability in human serum (% ± SD)	1 h	99.5 ± 0.2	99.7 ± 0.2	99.4 ± 0.2	97.9 ± 0.5	99.7 ± 0.3	98.2 ± 1.1
	2 h	99.5 ± 0.0	99.8 ± 0.1	99.8 ± 0.1	99.2 ± 0.6	99.8 ± 0.1	96.8 ± 0.5
	4 h	99.7 ± 0.1	99.6 ± 0.4	99.7 ± 0.2	98.9 ± 0.6	99.8 ± 0.2	96.9 ± 0.9

**Fig. 2:**
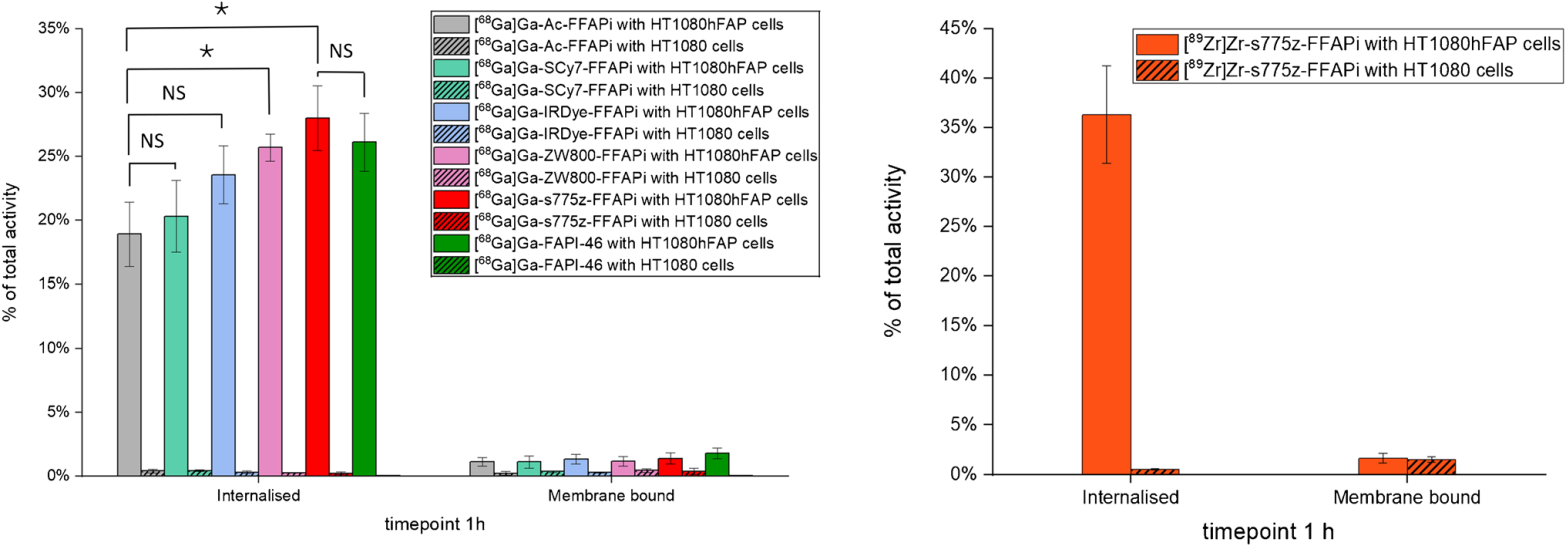
Cell-associated radioactivity determined after 1 h of incubation for the Gallium-68 labelled probes in HT1080hFAP and HT1080 cells (left) and analogous results for [^89^Zr]Zr-s775z-FFAPi (right). The final concentration of the radioligands was 1 nM per well. The values are reported as means of three independent experiments, with the exception of [^68^Ga]Ga-FAPI-46, for which data were obtained from a single experiment. The asterisks represent the level of significance determined by using the *p* value (*: 0.01 < *p* < 0.05; NS: not statistically significant)

**Fig. 3:**
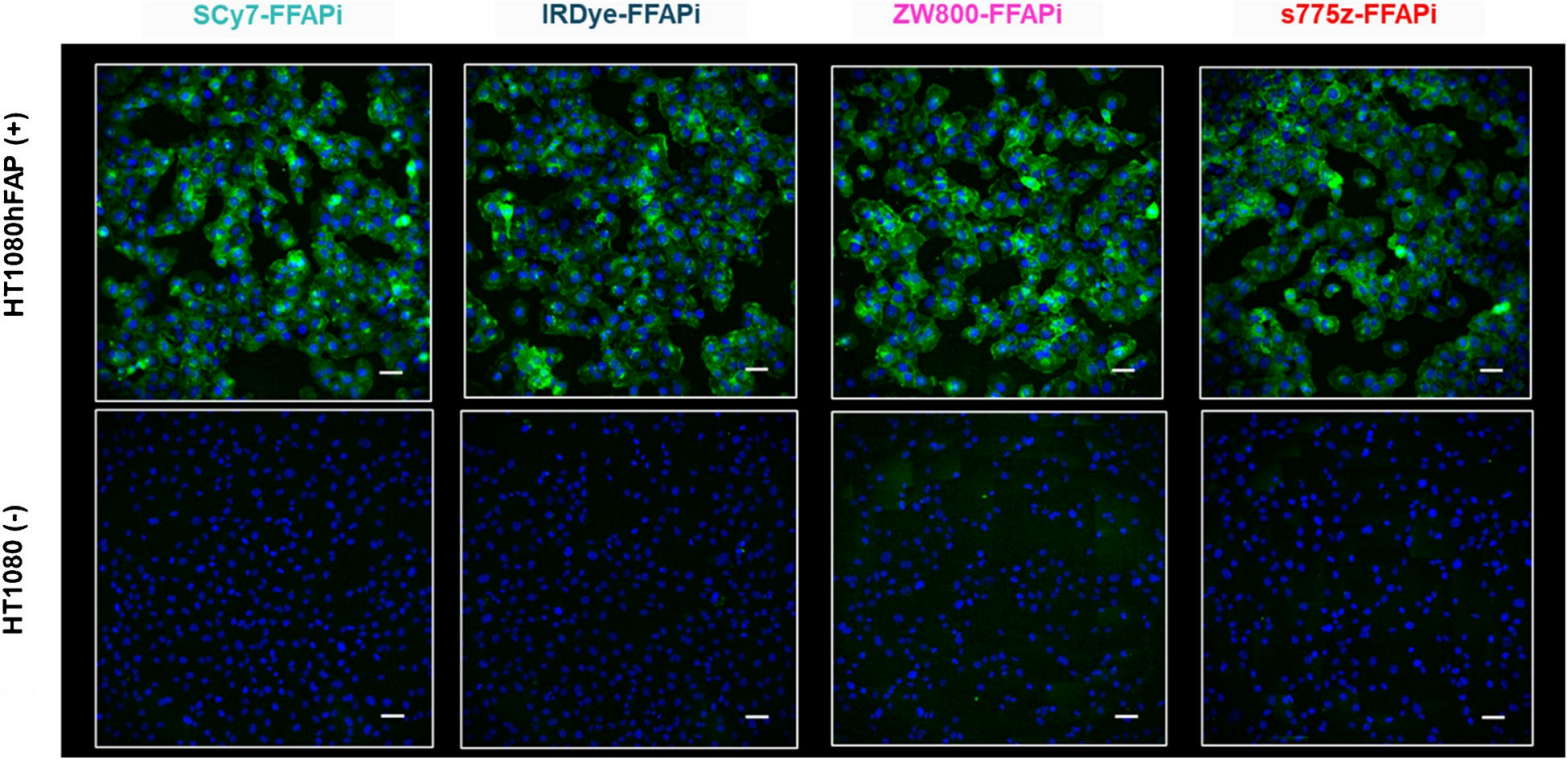
Cell-associated fluorescence of the non labelled dual-modality agents (final concentration: 500 nM/well) was measured after 1 h of incubation using HT1080hFAP and HT1080 cells. The images reported overlay the Cy7channel (green) showing the dual-modality agent and the HOECHST 3342 channel (blue) showing the nuclei. Magnification (20X) is equal for all images (scale bar: 50 µm)

Subsequently, we assessed the *in vitro* FAP-binding potential of the ligands through radioactive cell binding studies using FAP-expressing cells. All multimodal ligands exhibited specific uptake, with minimal levels detected in the membrane fraction (Fig. 2, left panel). Direct comparison revealed that [^68^Ga]Ga-ZW800-FFAPi and [^68^Ga]Ga-s775z-FFAPi showed the highest internalization rates in the group (25.7 ± 1.1% and 28.0 ± 2.5%, respectively). Notably, these values were statistically higher than those of the control (18.9 ± 2.5%), thereby demonstrating the beneficial influence of these fluorophores on cellular uptake. An analogous result to the Gallium-68 version was observed for the Zirconium-89-labelled counterpart of s775z-FFAPi using the same cellular model (Fig. 2, right panel). Confocal fluorescence microscopy further demonstrated cytosolic localization of the non-labelled bimodal compounds in HT1080hFAP cells, indicating FAP-mediated uptake for all candidates and corroborating previous findings with this model (Fig. 3)[40]. The FAP-affinity of the metal-bound [^nat^Ga]Ga-s775z-FFAPi and [^nat^Ga]Ga-IRDye-FFAPi was also evaluated, yelding IC50 values in the same nanomolar range (3.7–3.9 nM) as the reference [^nat^Ga]Ga-FAPI-46 (2.2 ± 0.2 nM; Table S1).

**Fig. 4:**
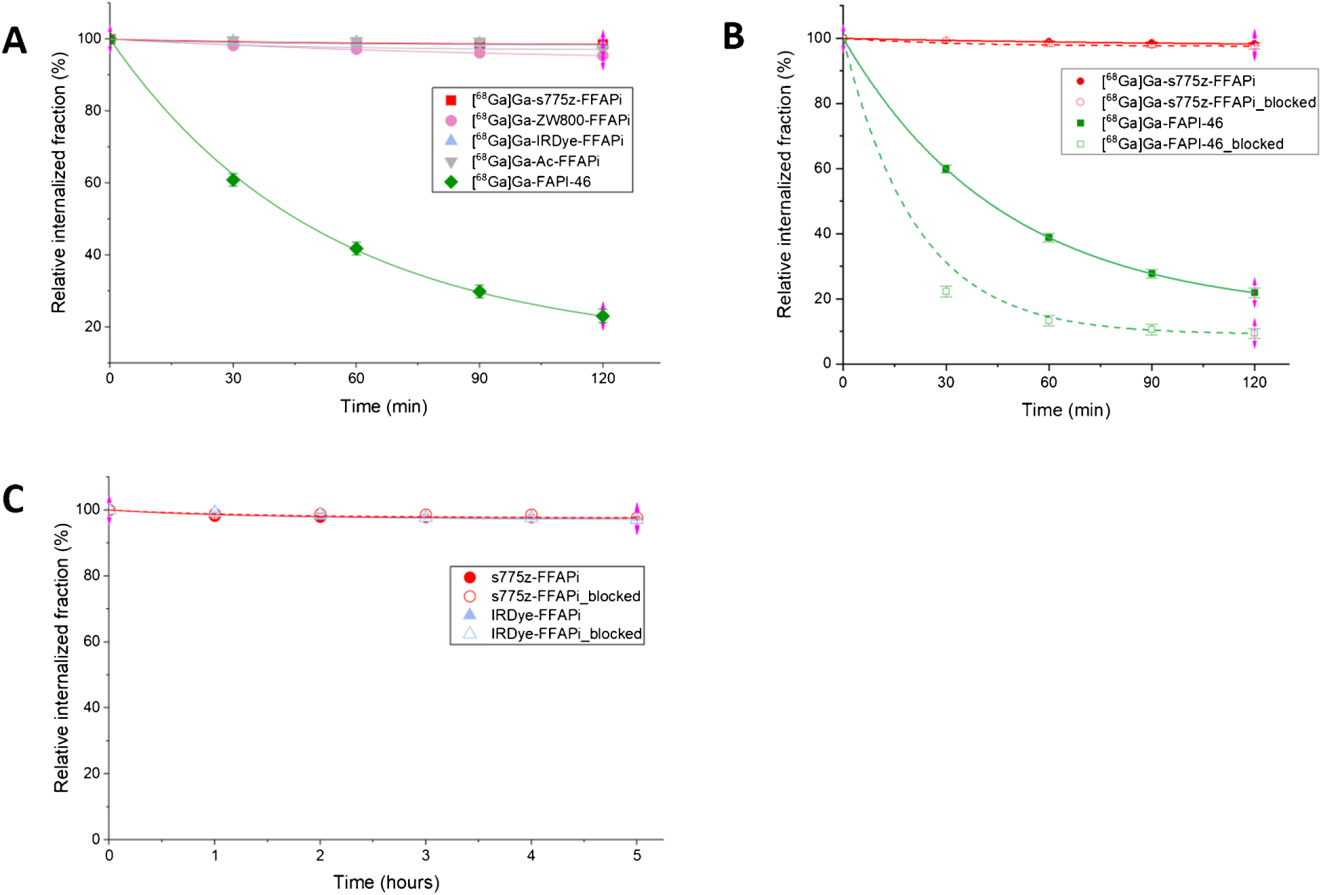
Cell-efflux study results from HT1080hFAP cells. Blocking conditions were obtained by incubating the cells in presence of culturing medium supplemented with FAPI-46 at a concentration of 1 µM. **A** Radioactive-based cell efflux of [^68^Ga]Ga-s775z-FFAPi, [^68^Ga]Ga-ZW800-FFAPi, [^68^Ga]Ga-IRDye-FFAPi, [^68^Ga]Ga-Ac-FFAPi and [^68^Ga]Ga-FAPI-46 up to 2 h after the end of the initial incubation at a concentration of 1 nM/well. All probes were tested in parallel in a head-to-head comparison study performed on the same day. Data are reported as means of three technical replicates wells from a single experiment. **B** Radioactive-based efflux of [^68^Ga]Ga-s775z-FFAPi and [^68^Ga]Ga-FAPI-46 up to 2 h after the end of the initial incubation at concentration of 1 nM/well. Data are presented as means of three independent experiments. **C** Fluorescence-based cell-efflux study of s775z-FFAPi and IRDye-FFAPi up to 5 h after the end of the initial incubation at a concentration of 20 nM/well. The probes were tested in parallel in a head-to-head comparison study performed on the same day. The values are reported as means of two technical replicate wells, each measured in triplicate

Cellular retention was evaluated by monitoring both the radioactive and fluorescence signals. As shown in Fig. 4A, the bimodal ligands and [^68^Ga]Ga-Ac-FFAPi maintained > 95% of the internalized fraction for up to 2 h post-initial incubation, with no evidence of significant washout. When the experiment was repeated with [^68^Ga]Ga-s775z-FFAPi and [^68^Ga]Ga-FAPi-46 under blocking conditions, designed to prevent re-binding of dissociated ligand, an effect was observed only for [^68^Ga]Ga-FAPi-46, where blocking lowered the retained activity (to 9.4 ± 1.5% of the internalized fraction under blocking vs. 21.9 ± 1.4% without blocking 2 h post-initial incubation; Fig. 4B). Cellular efflux was also assessed using fluorescence signals for s775z-FFAPi and IRDye-FFAPi, revealing trends consistent with the radioactive assays under both non-blocking and blocking conditions with < 4% release even 5 h post-initial incubation (Fig. 4C).

### *In vivo* evaluation

**Fig. 5:**
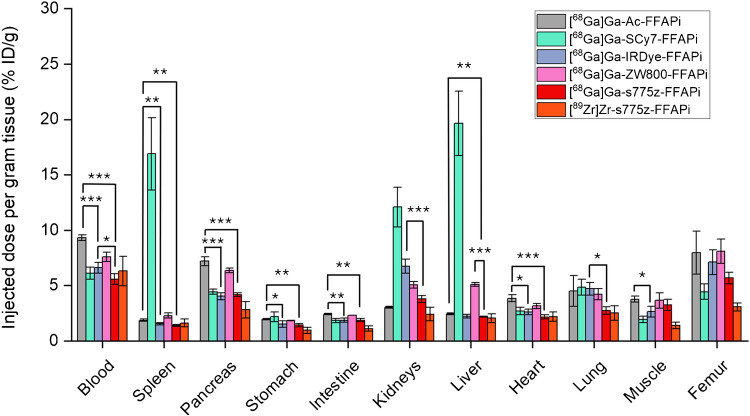
*Ex vivo* biodistribution studies in healthy BALB/C mice (n = 4) performed at 1 h p.i. for Gallium-68 labelled probes (amount injected: 0.10 nmol, 0.5 MBq) and for [^89^Zr]Zr-s775z-FFAPi (amount injected: 0.10 nmol, 0.08 MBq). The asterisks represent the level of significance determined by using the *p* value (*: 0.01 < *p* < 0.05; **: 0.001 < *p* < 0.01; ***: *p* < 0.001)

To obtain preliminary indications regarding the most promising candidates, we first investigated the biodistribution profile of the bimodal compounds through a head-to-head study in healthy mice injected with the same dose and activity of the different ligands (0.10 nmol, 0.5 MBq) (Fig. 5). Aside from the kidneys (%ID/g: 14.00 ± 1.79), [^68^Ga]Ga-SCy7-FFAPi exhibited out-of-trend uptake also in the spleen (%ID/g: 16.90 ± 3.27) and liver (%ID/g: 19.66 ± 2.91), suggesting the formation of aggregates.

[^68^Ga]Ga-s775z-FFAPi exhibited overall the most favorable profile in the group, showing lower accumulation in critical non-target organs, such as in the liver, when compared to [^68^Ga]Ga-ZW800-FFAPi (%ID/g: 2.20 ± 0.03 vs 5.10 ± 0.16), and in the kidneys when compared to [^68^Ga]Ga-IRDye-FFAPi (%ID/g: 3.78 ± 0.32 vs 6.75 ± 0.64). Notably, the direct comparison of [^68^Ga]Ga-s775z-FFAPi with [^68^Ga]Ga-Ac-FFAPi demonstrated that the introduction of the s775z fluorophore led to an overall improvement in the biodistribution profile, as evidenced by the statistically lower levels in most organs for the s775z-derivative – including blood, spleen, pancreas, stomach, intestine, liver, and heart (Fig. 5 and Table S2). When labelled with Zirconium-89, s775z-FFAPi exhibited a biodistribution profile similar to its Gallium-68 counterpart, with slightly reduced accumulation in the pancreas, stomach, intestine, kidneys, muscle and femur (Table S3). Furthermore, no evidence of *in vivo* degradation was detected for [^68^Ga]Ga-s775z-FFAPi both in serum or urine at 15 min p.i. (Fig. S33), whereas only minor signs were noticed for [^68^Ga]Ga-IRDye-FFAPi (Fig. S34). Analogously to the s775z one, the IRDye-derivative also exhibited significantly lower levels in multiple organs when compared to the control compound -including blood, spleen, pancreas, stomach, intestine, heart and muscle- suggesting that the IRDye800CW similarly contributes to improved biodistribution (Fig. 5). Finally, when directly compared to [^68^Ga]Ga-IRDye-FFAPi, [^68^Ga]Ga-s775z-FFAPi showed statistically lower uptake in the kidney, blood, and lung.

**Fig. 6:**
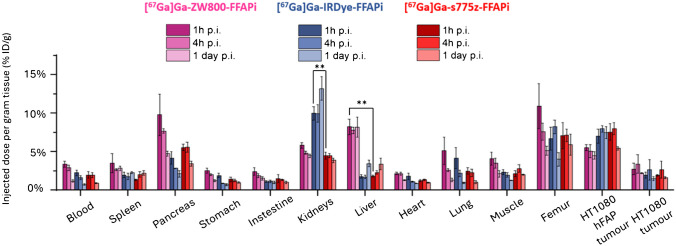
*Ex vivo* biodistribution studies in HT1080hFAP/HT1080 xenografted BALB/C nude mice (*n* = 3) performed for 1 h, 4 h and 1 day p.i. for Gallium-67 labelled probes (amount injected: 0.25 nmol, 0.4 MBq). The asterisks represent the level of significance determined by using the *p* value (*: 0.01 < *p* < 0.05; **: 0.001 < *p* < 0.01; ***: *p* < 0.001)

In a subsequent phase of the project, we evaluated the *in vivo* targeting and tumor retention of the Gallium-67 labelled ZW800-FFAPi, IRDye-FFAPi and s775z-FFAPi up to 1 day post-injection (p.i.) in HT1080hFAP/HT1080 xenografted mice (Fig. 6). All multimodal ligands demonstrated specific accumulation in the FAP-expressing tumor. As shown in Table S4A-C, at 1 h post-injection (p.i.), [^67^Ga]Ga-s775z-FFAPi and [^67^Ga]Ga-IRDye-FFAPi exhibited tumor uptake values of 7.57 ± 1.06 and 7.04 ± 0.91%ID/g, respectively, which were not statistically different from that of [^67^Ga]Ga-ZW800-FFAPi (5.44 ± 0.38%ID/g; *p* > 0.05). At 1 h p.i. the hFAP-positive to hFAP-negative tumor ratios were 4.34 ± 0.18, 3.61 ± 1.60 and 2.04 ± 0.67, respectively. Notably, all the tracers displayed good tumor retention at 1 day p.i., with uptake values of 5.44 ± 0.23%ID/g for [^67^Ga]Ga-s775z-FFAPi, 7.48 ± 0.79%ID/g for [^67^Ga]Ga-IRDye-FFAPi and 4.51 ± 0.49%ID/g for [^67^Ga]Ga-ZW800-FFAPi. The corresponding hFAP-positive to hFAP-negative tumor ratios at this time point were 3.42 ± 0.59, 4.75 ± 1.16 and 1.88 ± 0.12, respectively.

Compared to [^67^Ga]Ga-ZW800-FFAPi (1.41 ± 0.32), [^67^Ga]Ga-s775z-FFAPi and [^67^Ga]Ga-IRDye-FFAPi exhibited significantly higher hFAP-positive tumor to muscle ratios at 1 h p.i. (3.76 ± 1.40, *p* = 0.047 and 3.05 ± 0.20, *p* = 0.0017). Interestingly, this ratio decreased to 2.72 ± 0.22 for the s775z derivative at 1 day p.i., while it increased to 6.05 ± 0.69 for the IRDye-800CW conjugate, indicating better contrast for this candidate at later time points (*p* = 0.0013). When assessed based on fluorescence signal, tumor-to-muscle ratios greater than 4 were observed for these candidates at both 1 h and 1 day post-injection (Fig. S35-36).

Regarding accumulation in non-target organs, when compared to [^67^Ga]Ga-s775z-FFAPi, [^67^Ga]Ga-IRDye-FFAPi exhibited markedly higher renal accumulation (*p* = 0.0013), whereas [^67^Ga]Ga-ZW800-FFAPi displayed distinct higher hepatic accumulation (*p* = 0.0010).

**Fig. 7:**
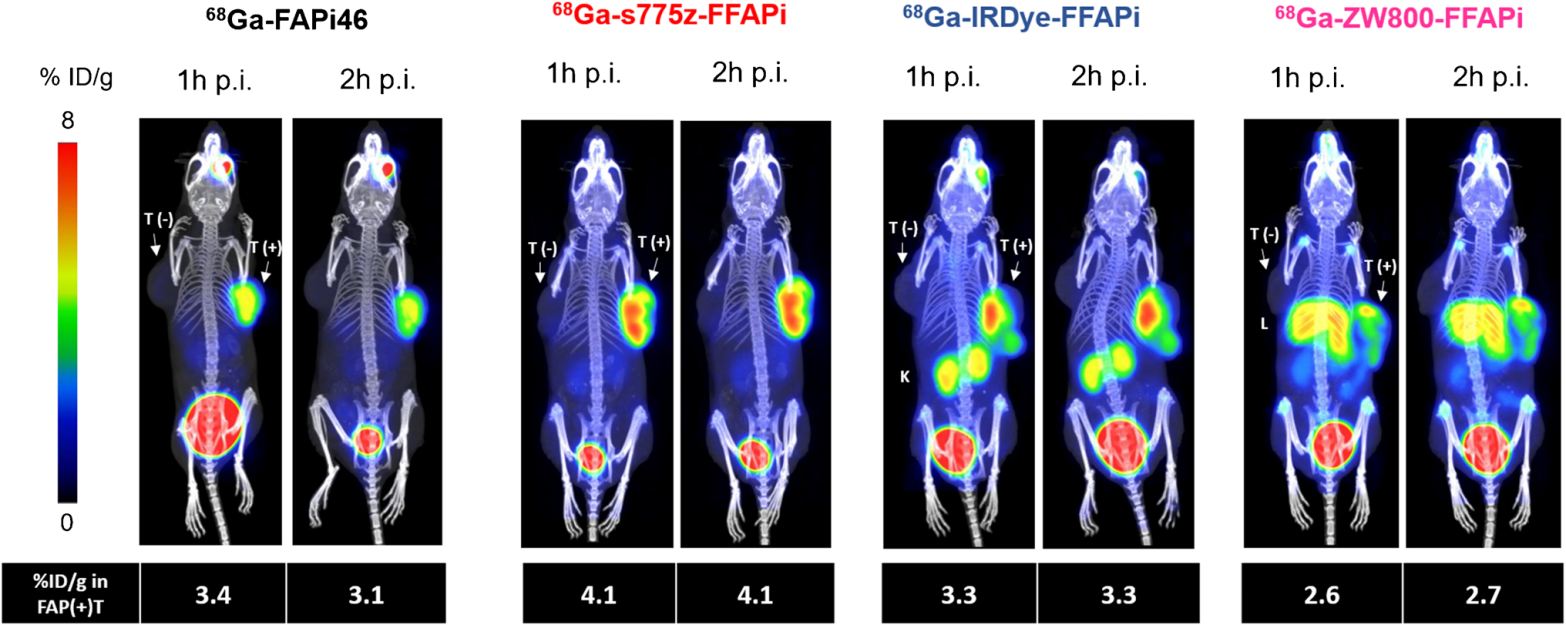
Static PET/CT MIP images of HT1080hFAP/HT1080 xenografted BALB/C nude mice injected with Gallium-68 labelled dual-modality agents and [^68^Ga]Ga-FAPI-46 as reference (amount injected: 1.0 nmol, 5.0–8.0 MBq). L = liver, K = kidney, T(+) = HT1080hFAP tumor, T(-) = HT1080 tumor. One mouse was imaged per compound. Below each image, the uptake in the HT1080hFAP tumor is expressed as the mean percentage of the injected dose per gram (%ID/g). Values were calculated using manually drawn ROIs fitting to the tumor

PET/CT imaging at early time points with the Gallium-68-labelled versions of the three tracers confirmed the differences observed in the biodistribution studies (Fig. 7). Among the evaluated bimodal tracers, [^68^Ga]Ga-s775z-FFAPi emerged as the most promising candidate. Additionally, based on PET data from a single animal per compound, [^68^Ga]Ga-s775z-FFAPi suggested an image quality at this early time point that was comparable to that of the clinically established [^68^Ga]Ga-FAPi-46.

The quantitative biodistribution results obtained at 1 h p.i. (Table S4) showed good agreement with the qualitative evaluation of the fluorescence emitted from the same organs for this early time point (Fig. S35).

**Fig. 8:**
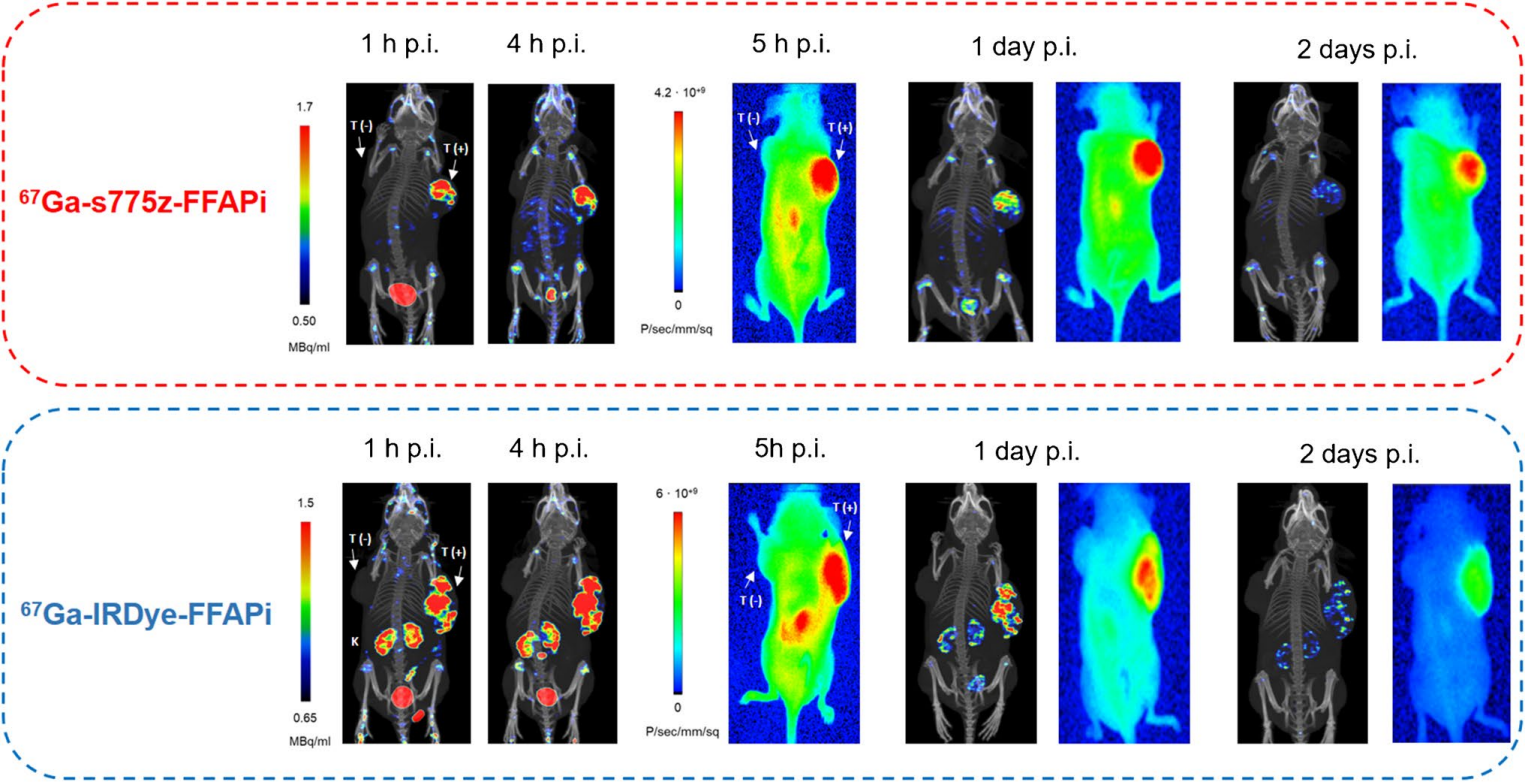
Static SPECT/CT MIP images and corresponding near-infrared fluorescence images were acquired at various time points for HT1080hFAP/HT1080 xenografted BALB/C nude mice (one per compound) injected with [^67^Ga]Ga-s775z-FFAPi or with [^67^Ga]Ga-IRDye-FFAPi (1.5–1.8 nmol, 15–17 MBq)

To evaluate the potential of our ligands for intraoperative radio- and fluorescence-guided surgery also for later time points, we injected xenografted mice with Gallium-67 labelled s775z-FFAPi and IRDye-FFAPi and imaged them using an animal SPECT/CT and near-infrared fluorescence (NIRF) system (Fig. 8). The subcutaneous HT1080hFAP-positive tumors were clearly visualized with these candidates using both imaging modalities up to 1 day p.i. By 2 days p.i., satisfactory tumor visualization was achieved only through the fluorescent signal.

## Discussion

Incomplete surgical resection remains common, can lead to avoidable additional therapies and may negatively impact prognosis [41]. Fluorescence-guided surgery (FGS) offers a promising approach to improve surgical precision, particularly in head and neck squamous cell carcinoma (HNSCC) [7]. The fibroblast activation protein (FAP) has emerged as a valuable target due to its high expression in cancer-associated fibroblasts (CAFs) across many tumor types, including HNSCC. To bridge preoperative tumor evaluation with intraoperative surgical guidance, the first reported dual-modality imaging agents targeting FAP, [^68^Ga]Ga-FAP-2286-ICG and [^18^F]F-NOTA-FAPI-MB, have recently been investigated [21, 22]. Although the incorporation of the respective fluorophores preserved *in vitro* performance, they negatively altered the *in vivo* biodistribution, leading to suboptimal tumor-to-background contrast. Therefore, these preliminary studies underscored the investigation of alternative fluorophores that can be integrated into a FAPI radiotracer without significantly affecting its original pharmacokinetics. In parallel to this study, Zhao, Liang. et al. used IRDye-800CW with considerable improvement in pharmacokinetics similar to the unmodified peptide based FAP ligands (FAP-2286 and FAP 3BP-3940) [25].

Since the clinical potential of a dual-modality imaging agent would be greater if both pre- and intraoperative imaging were achievable with a single injection, we focused on developing probes with extended tumor retention. Inspired by the encouraging results obtained with FAPI homodimers, we explored whether applying the multivalency concept could be a promising strategy for dual-modality probes [26, 27].

The multifunctional chelator Fusarinine C (FSC) has already demonstrated its feasibility as a scaffold for developing multimers, including homodimeric dual-modality agents [42–45]. Besides Gallium-68 it also allows radiolabelling with Zirconium-89, which potentially enables decentralized production for PET imaging. Therefore, we selected FSC for this study. The resulting probes incorporated two FAP-binding motifs, connected through PEG4 linkers for enhanced solubility, along with one of four near-infrared (NIR) fluorophores from the cyanine heptamethine dye family (SulfoCy7, IRDye800CW, ZW800 and s775z), selected for their differing physicochemical properties. To the best of our knowledge, the reported s775z-FFAPi ligand represents the first example of conjugating s775z to a small molecule—specifically, a FAP-targeting ligand—and its first application in dual-modality imaging.

A variety of cellular models are currently employed to evaluate FAP-targeted tracers, with primary human CAFs being among the most translationally relevant. However, their limited availability and inter-patient variability often necessitate the use of more standardized alternatives. Recently, Van der Heide et al. evaluated several preclinical models, including FAP-transduced cancer cells and cell lines with endogenous FAP expression [40]. They concluded that, while all models are only approximations of the clinical scenario, they differ in their ability to reproduce both the expression levels of FAP and the heterogeneous, stromal-specific expression patterns observed in patients, with the latter being more clinically relevant. Despite these considerations, we employed the HT1080h-FAP transduced cell line, a reproducible model well suited for screening the developed ligands in terms of target binding, specificity, retention, and imaging performance. In addition, it remains one of the most widely used FAP-expressing models in preclinical research, thereby enabling comparison across studies. To further facilitate this, [^68^Ga]Ga-FAPI-46 was also included as an internal reference in the main experiments.

No conclusive correlation was identified between lipophilicity, protein affinity and cellular uptake across the probes* (*see discussion in Supplementary Material*). Ex vivo* and *in vivo* studies revealed distinct biodistribution profiles depending on the fluorophore. SCy7-FFAPi accumulated in the spleen and liver, likely due to aggregation associated with its higher lipophilicity (Fig. 5). Pronounced renal accumulation was observed for [^67^Ga]Ga-IRDye-FFAPi, a behavior characteristic of IRDye800CW derivatives and presumably attributable to the sulfonic group located on a flexible arm attached to one of the indole rings, as previously reported (Fig. 5–8) [32]. By contrast, [^67^Ga]Ga-ZW800-FFAPi exhibited notable hepatic accumulation, which could be related to the reported instability of the ether bond *in vivo* [46, 47].

While no direct comparison with [^67^Ga]Ga-FAPI-46 was performed, the observed *in vivo* tumor retention aligns with the* in vitro* data and reflects the influence of dimerization, as shown by the comparison between [^68^Ga]Ga-Ac-FFAPi and [^68^Ga]Ga-FAPi-46 (Fig. 4A). This retention was also confirmed for the fluorescent label (Fig. 4C). *Ex vivo* analysis at 1 day p.i. showed tumor retention rates of 72% for [^67^Ga]Ga-s775-FFAPi, 81% for [^67^Ga]Ga-ZW800-FFAPi, and full retention for [^67^Ga]Ga-IRDye-FFAPi, relative to the levels measured at 1 h p.i. (Fig. 6). Additionally, the SPECT/CT images obtained with [^67^Ga]Ga-s775z-FFAPi and [^67^Ga]Ga-IRDye-FFAPi suggested that the biodistribution profiles achievable at early time points were overall preserved for later time points (Fig. 8). A careful evaluation of the radioactive levels for these probes in resected organs during *ex vivo* biodistribution experiments indicated an approximate twofold increase of liver uptake from 1 h to 1 day p.i., still with final levels remaining below 4% ID/g (Table S4B-C). This trend matched the rise in fluorescent signal in the optical biodistribution study (Fig. S35) and resulted in decreased FAP(+)-tumor-to-liver ratio (Fig. S36).

Noteworthy, we demonstrated that the introduction of the s775z fluorophore had favorable impact on various tracer properties. The direct comparison between [^68^Ga]Ga-s775z-FFAPi and [^68^Ga]Ga-Ac-FFAPi revealed that, despite being the bulkiest modification to the scaffold, s775z increased both hydrophilicity and cellular internalization. *In vivo*, s775z incorporation overall improved the biodistribution profile of the original scaffold, as indicated by the lower accumulation in blood and most organs for [^68^Ga]Ga-s775z-FFAPi compared to [^68^Ga]Ga-Ac-FFAPi (Fig. 5). Among all tested bimodal candidates, [^68^Ga]Ga-s775z-FFAPi exhibited the most favorable profile, particularly by minimizing renal and hepatic off-target accumulation (Fig. 7). This observation differentiates this compound also from other reported heptamethine dye-based analogues and can be attributed to the specific structural features of this dye [21, 25].

To our knowledge, the simultaneous beneficial impact of dye incorporation on both the *in vitro* properties and *in vivo* pharmacokinetics of a dual-modality imaging agent has not previously been reported for FAP-targeted ligands. However, for αvβ3-targeting ligands, Bunschoten et al. screened nine pentamethine dye candidates with different geometrical substitutions and net charges, ultimately identifying a single variant whose incorporation preserved receptor affinity while imparting overall improved *in vivo* characteristics compared to the fluorophore-free scaffold [32].

In the case of Prostate-Specific Membrane Antigen (PSMA)-targeting agents, Baranski et al. reported increased cellular internalization and tumor uptake following the incorporation of IRDye800CW or DyLight800. Similarly, Derks et al. observed enhanced cellular binding upon incorporation of IRDye700DX. Additionally in this case, direct comparison of [^111^In]In-N064 with the control compound lacking the fluorophore demonstrated preservation of the biodistribution profile, with the sole exception of the kidneys [33, 48].

The IRDye800CW dye, although to a lesser extent than s775z, also positively impacted the biodistribution profile, leading to reduced off-target accumulation in various organs (Fig. 5 and Table S2). Although [^67^Ga]Ga-IRDye-FFAPi showed higher kidney accumulation and retention than the s775z analog, it exhibited superior tumor uptake at later time points resulting in higher tumor-to-muscle contrast. Therefore, at this stage both compounds showed comparable translational potential and warrant further investigation—both in studies assessing their binding to samples with clinically relevant FAP expression and in experiments evaluating their applicability in a real surgical setting.

## Conclusions

In this preclinical study, we developed dimeric dual-modality imaging agents targeting FAP for the first time. In addition, we systematically investigated the impact of different heptamethine cyanine fluorophores on the *in vitro* and *in vivo *properties of these agents.

Notably, after a single administration, s775z-FFAPi and IRDye-FFAPi demonstrated rapid clearance from healthy organs and selective accumulation in tumor tissue, as observed simultaneously by SPECT and fluorescence imaging in the same animals. This accumulation persisted for a duration suitable for the intended application and provided satisfactory tumor-to-background ratios. Overall, this work emphasizes that through careful molecular design, targeted dual-modality agents with significant potential for preoperative detection and intraoperative margin delineation of FAP-positive malignancies can be developed.

## Supplementary Information

Below is the link to the electronic supplementary material.

Supplementary file1 (DOCX 7.66 MB)

## Data Availability

The datasets generated and/or analysed during the current study are available from the corresponding author on reasonable request.

## References

[1] Johnson DE, Burtness B, Leemans CR, Lui VWY, Bauman JE, Grandis JR. Head and neck squamous cell carcinoma. Nat Rev Dis Primers. 2020;6:92. 33243986 10.1038/s41572-020-00224-3 PMC7944998

[2] National Comprehensive Cancer Network. NCCN Guidelines for Patients: Mouth Cancers. In: NCCN Guidelines for Patients: Head and Neck Cancers. National Comprehensive Cancer Network; 2024. Available from: https://www.nccn.org/patients/guidelines/content/PDF/hn-oral-patient.pdf. Accessed 30 Oct 2025.

[3] Kröplin J, Reppenhagen J-C. Best practices and future challenges in the treatment of oral cancer. Innovative Surgical Sciences. 2024;8:215–20. 38510366 10.1515/iss-2023-0031 PMC10949209

[4] Castaldi P, Leccisotti L, Bussu F, Miccichè F, Rufini V. Role of (18)F-FDG PET-CT in head and neck squamous cell carcinoma. Acta Otorhinolaryngol Ital. 2013;33:1–8. 23620633 PMC3631810

[5] Rosenthal EL, Warram JM, De Boer E, Chung TK, Korb ML, Brandwein-Gensler M, et al. Safety and tumor specificity of cetuximab-IRDye800 for surgical navigation in head and neck cancer. Clin Cancer Res. 2015;21:3658–66. 25904751 10.1158/1078-0432.CCR-14-3284 PMC4909371

[6] Rosenthal EL, Moore LS, Tipirneni K, de Boer E, Stevens TM, Hartman YE, et al. Sensitivity and specificity of cetuximab-IRDye800CW to identify regional metastatic disease in head and neck cancer. Clin Cancer Res. 2017;23:4744–52. 28446503 10.1158/1078-0432.CCR-16-2968 PMC5595145

[7] van Keulen S, Nishio N, Fakurnejad S, Birkeland A, Martin BA, Lu G, et al. The clinical application of fluorescence-guided surgery in head and neck cancer. J Nucl Med. 2019;60:758–63. 30733319 10.2967/jnumed.118.222810 PMC6581234

[8] Kubeil M, Martínez IIS, Bachmann M, Kopka K, Tuck KL, Stephan H. Dual-labelling strategies for nuclear and fluorescence molecular imaging: current status and future perspectives. Pharmaceuticals. 2022;15:432. 35455430 10.3390/ph15040432 PMC9028399

[9] Zhao J, Chen J, Ma S, Liu Q, Huang L, Chen X, et al. Recent developments in multimodality fluorescence imaging probes. Acta Pharm Sin B. 2018;8:320–38. 29881672 10.1016/j.apsb.2018.03.010 PMC5989919

[10] Zi F, He J, He D, Li Y, Yang L, Cai Z. Fibroblast activation protein α in tumor microenvironment: recent progression and implications. Mol Med Rep. 2015;11:3203–11. 25593080 10.3892/mmr.2015.3197 PMC4368076

[11] Kratochwil C, Flechsig P, Lindner T, Abderrahim L, Altmann A, Mier W, et al. ^68^Ga-FAPI PET/CT: tracer uptake in 28 different kinds of cancer. J Nucl Med. 2019;60:801–5. 30954939 10.2967/jnumed.119.227967 PMC6581228

[12] Lindner T, Loktev A, Altmann A, Giesel F, Kratochwil C, Debus J, et al. Development of quinoline-based theranostic ligands for the targeting of fibroblast activation protein. J Nucl Med. 2018;59:1415–22. 29626119 10.2967/jnumed.118.210443

[13] Li D, Li X, Zhao J, Tan F. Advances in nuclear medicine-based molecular imaging in head and neck squamous cell carcinoma. J Transl Med. 2022;20:358. 35962347 10.1186/s12967-022-03559-5 PMC9373390

[14] Mitha M, Aden D, Zaheer S, Alvi Y. Role of tumor budding and fibrotic cancer stroma in head and neck squamous cell carcinoma. Pathol-Res Pract. 2024;253:155052. 38176309 10.1016/j.prp.2023.155052

[15] Cacchi C, Fischer HJ, Wermker K, Rashad A, Jonigk DD, Hölzle F, et al. New tumor budding evaluation in head and neck squamous cell carcinomas. Cancers (Basel). 2024;16:587. 38339338 10.3390/cancers16030587 PMC10854693

[16] Dourado MR, Miwa KY, Hamada GB, Paranaiba LM, Sawazaki-Calone Í, Domingueti CB, et al. Prognostication for oral squamous cell carcinoma patients based on the tumour–stroma ratio and tumour budding. Histopathology. 2020;76:906–18. 31984527 10.1111/his.14070

[17] Rizzo R, Capozza M, Conti L, Avalle L, Poli V, Terreno E. Novel FAP-targeted heptamethine cyanines for NIRF imaging applications. Mol Pharm. 2025. 10.1021/acs.molpharmaceut.4c01232. 39954291 10.1021/acs.molpharmaceut.4c01232 PMC11881144

[18] Roy J, Hettiarachchi SU, Kaake M, Mukkamala R, Low PS. Design and validation of fibroblast activation protein alpha targeted imaging and therapeutic agents. Theranostics. 2020;10:5778. 32483418 10.7150/thno.41409 PMC7254991

[19] Slania SL, Das D, Lisok A, Du Y, Jiang Z, Mease RC, et al. Imaging of fibroblast activation protein in cancer xenografts using novel (4-quinolinoyl)-glycyl-2-cyanopyrrolidine-based small molecules. J Med Chem. 2021;64:4059–70. 33730493 10.1021/acs.jmedchem.0c02171 PMC8214312

[20] Lin E, Song M, Wang B, Shi X, Zhao J, Fu L, et al. Fibroblast activation protein peptide-targeted NIR-I/II fluorescence imaging for stable and functional detection of hepatocellular carcinoma. Eur J Nucl Med Mol Imaging. 2025. 10.1007/s00259-025-07093-6. 39836214 10.1007/s00259-025-07093-6

[21] Li D, Li X, Li J, Wang Y, Tan F, Li X. Development of a fibroblast activation protein-targeted PET/NIR dual-modality probe and its application in head and neck cancer. Front Bioeng Biotechnol. 2023;11:1291824. 38026901 10.3389/fbioe.2023.1291824 PMC10654779

[22] Zhao X, Zhang G, Chen J, Li Z, Shi Y, Li G, et al. A rationally designed nuclei-targeting FAPI 04-based molecular probe with enhanced tumor uptake for PET/CT and fluorescence imaging. Eur J Nucl Med Mol Imaging. 2024;51:1593–604. 38512485 10.1007/s00259-024-06691-0

[23] Zhang X, Huang J, Gong F, Cai Z, Liu Y, Tang G, et al. Synthesis and preclinical evaluation of a novel PET/fluorescence dual-modality probe targeting fibroblast activation protein. Bioorg Chem. 2024;146:107275. 38493637 10.1016/j.bioorg.2024.107275

[24] Ariztia J, Solmont K, Moise NP, Specklin S, Heck MP, Lamande-Langle S, et al. PET/fluorescence imaging: an overview of the chemical strategies to build dual imaging tools. Bioconjug Chem. 2022;33:24–52. 34994545 10.1021/acs.bioconjchem.1c00503

[25] Zhao L, Pang Y, Xu D, Chen J, Yu S, Ruan D, et al. Preclinical and pilot clinical evaluation of novel dual-modality pet/fluorescence probes targeting FAP for accurate tumor margin delineation. Eur J Nucl Med Mol Imaging. 2025;1–12. 10.1007/s00259-025-07512-8 40848055

[26] Ballal S, Yadav MP, Moon ES, Kramer VS, Roesch F, Kumari S, et al. First-in-human results on the biodistribution, pharmacokinetics, and dosimetry of [177Lu] Lu-DOTA. SA. FAPi and [177Lu] Lu-DOTAGA. (SA. FAPi) 2. Pharmaceuticals. 2021;14:1212. 34959613 10.3390/ph14121212 PMC8707268

[27] Galbiati A, Zana A, Bocci M, Millul J, Elsayed A, Mock J, et al. A dimeric FAP-targeting small-molecule radioconjugate with high and prolonged tumor uptake. J Nucl Med. 2022;63:1852–8. 35589404 10.2967/jnumed.122.264036 PMC9730928

[28] Choi HS, Gibbs SL, Lee JH, Kim SH, Ashitate Y, Liu F, et al. Targeted zwitterionic near-infrared fluorophores for improved optical imaging. Nat Biotechnol. 2013;31:148–53. 23292608 10.1038/nbt.2468 PMC3568187

[29] Usama SM, Thapaliya ER, Luciano MP, Schnermann MJ. Not so innocent: impact of fluorophore chemistry on the in vivo properties of bioconjugates. Curr Opin Chem Biol. 2021;63:38–45. 33684856 10.1016/j.cbpa.2021.01.009 PMC8384635

[30] Debie P, Hernot S. Emerging fluorescent molecular tracers to guide intra-operative surgical decision-making. Front Pharmacol. 2019;10:510. 31139085 10.3389/fphar.2019.00510 PMC6527780

[31] Hensbergen AW, Buckle T, van Willigen DM, Schottelius M, Welling MM, van der Wijk FA, et al. Hybrid tracers based on cyanine backbones targeting prostate-specific membrane antigen: tuning pharmacokinetic properties and exploring dye–protein interaction. J Nucl Med. 2020;61:234–41. 31481575 10.2967/jnumed.119.233064 PMC8801960

[32] Bunschoten A, van Willigen DM, Buckle T, van den Berg NS, Welling MM, Spa SJ, et al. Tailoring fluorescent dyes to optimize a hybrid RGD-tracer. Bioconjug Chem. 2016;27:1253–8. 27074375 10.1021/acs.bioconjchem.6b00093

[33] Baranski A-C, Schäfer M, Bauder-Wüst U, Roscher M, Schmidt J, Stenau E, et al. PSMA-11–derived dual-labeled PSMA inhibitors for preoperative PET imaging and precise fluorescence-guided surgery of prostate cancer. J Nucl Med. 2018;59:639–45. 29191856 10.2967/jnumed.117.201293

[34] Buckle T, van Willigen DM, Spa SJ, Hensbergen AW, van der Wal S, de Korne CM, et al. Tracers for fluorescence-guided surgery: how elongation of the polymethine chain in cyanine dyes alters the pharmacokinetics of a dual-modality c [RGDyK] tracer. J Nucl Med. 2018;59(6):986–92. 29449447 10.2967/jnumed.117.205575

[35] Gamage RS, Li D-H, Schreiber CL, Smith BD. Comparison of cRGDfK peptide probes with appended shielded heptamethine cyanine dye (s775z) for near infrared fluorescence imaging of cancer. ACS Omega. 2021;6:30130–9. 34778684 10.1021/acsomega.1c04991 PMC8582267

[36] Li DH, Schreiber CL, Smith BD. Sterically shielded heptamethine cyanine dyes for bioconjugation and high performance near-infrared fluorescence imaging. Angew Chem. 2020;132:12252–9. 10.1002/anie.202004449 PMC7473488 32324959

[37] Schindelin J, Arganda-Carreras I, Frise E, Kaynig V, Longair M, Pietzsch T, et al. Fiji: an open-source platform for biological-image analysis. Nat Methods. 2012;9:676–82. 22743772 10.1038/nmeth.2019 PMC3855844

[38] Schrettl M, Bignell E, Kragl C, Sabiha Y, Loss O, Eisendle M, et al. Distinct roles for intra-and extracellular siderophores during *Aspergillus fumigatus* infection. PLoS Pathog. 2007;3:e128. 17845073 10.1371/journal.ppat.0030128 PMC1971116

[39] Toms J, Kogler J, Maschauer S, Daniel C, Schmidkonz C, Kuwert T, et al. Targeting fibroblast activation protein: radiosynthesis and preclinical evaluation of an ^18^F-labeled FAP inhibitor. J Nucl Med. 2020;61:1806–13. 32332144 10.2967/jnumed.120.242958

[40] van der Heide CD, Campeiro JD, Ruigrok EA, van den Brink L, Ponnala S, Hillier SM, et al. In vitro and ex vivo evaluation of preclinical models for FAP-targeted theranostics: differences and relevance for radiotracer evaluation. EJNMMI Res. 2024;14:125. 39718718 10.1186/s13550-024-01191-6 PMC11668701

[41] Orosco RK, Tapia VJ, Califano JA, Clary B, Cohen EE, Kane C, et al. Positive surgical margins in the 10 most common solid cancers. Sci Rep. 2018;8:5686. 29632347 10.1038/s41598-018-23403-5 PMC5890246

[42] Summer D, Rangger C, Klingler M, Laverman P, Franssen GM, Lechner BE, et al. Exploiting the concept of multivalency with ^68^Ga- and ^89^Zr-labelled fusarinine C-minigastrin bioconjugates for targeting CCK2R expression. Contrast Media Mol Imaging. 2018. 10.1155/2018/3171794. 29849512 10.1155/2018/3171794 PMC5914118

[43] Zhai C, Summer D, Rangger C, Franssen GM, Laverman P, Haas H, et al. Novel bifunctional cyclic chelator for ^89^Zr labeling-radiolabeling and targeting properties of RGD conjugates. Mol Pharm. 2015. 10.1021/acs.molpharmaceut.5b00128. 25941834 10.1021/acs.molpharmaceut.5b00128 PMC4453016

[44] Summer D, Grossrubatscher L, Petrik M, Michalcikova T, Novy Z, Rangger C, et al. Developing targeted hybrid imaging probes by chelator scaffolding. Bioconjug Chem. 2017;28:1722–33. 28462989 10.1021/acs.bioconjchem.7b00182 PMC5481817

[45] Gariglio G, Bendova K, Hermann M, Olafsdottir A, Sosabowski JK, Petrik M, et al. Comparison of two chelator scaffolds as basis for cholecystokinin-2 receptor targeting bimodal imaging probes. Pharmaceuticals. 2024;17:1569. 39770411 10.3390/ph17121569 PMC11676163

[46] Hyun H, Owens EA, Narayana L, Wada H, Gravier J, Bao K, et al. Central c-c bonding increases optical and chemical stability of NIR fluorophores. RSC Adv. 2014;4:58762–8. 25530846 10.1039/C4RA11225C PMC4267294

[47] Su D, Teoh CL, Samanta A, Kang N-Y, Park S-J, Chang Y-T. The development of a highly photostable and chemically stable zwitterionic near-infrared dye for imaging applications. Chem Commun. 2015;51:3989–92. 10.1039/c4cc08814j 25664357

[48] Derks YH, Rijpkema M, Amatdjais-Groenen HI, Kip A, Franssen GM, Sedelaar JM, et al. Photosensitizer-based multimodal PSMA-targeting ligands for intraoperative detection of prostate cancer. Theranostics. 2021;11:1527. 33408764 10.7150/thno.52166 PMC7778589

