# Supplementary material for: Novel dimeric dual-modality FAP-targeted agents with favorable tumor retention for image-guided surgery: a preclinical study
Source: Eur J Nucl Med Mol Imaging. Author manuscript; Available in PMC 2025 Dec 2. (PMC12666486; doi:10.1007/s00259-025-07626-z)
Supplement: Supplementary file 1 [file NIHMS2124370-supplement-supp_material.docx]

**Supplementary Material**

**Novel dimeric dual-modality FAP-targeted agents with favourable tumor retention for image-guided surgery: a preclinical study**

Giacomo Gariglio ^1^, Thomas Hasenöhrl ^2^, Katerina Bendova ^3^, Zbyněk Nový ^3^, Christine Rangger ^1^, Kai Kummer ^4^, Bradley D. Smith^ 5^, Barbara Matuszczak ^2^, Milos Petrik ^3 ^and Clemens Decristoforo ^1,^*

^1^	Department of Nuclear Medicine, Medical University of Innsbruck, 6020 Innsbruck, Austria

^2^	Institute of Pharmacy, Department of Pharmaceutical Chemistry, University of Innsbruck, 6020 Innsbruck, Austria

^3^	Institute of Molecular and Translational Medicine, Faculty of Medicine and Dentistry and Czech Advanced Technology and Research Institute, Palacky University, 77900 Olomouc, Czech Republic

^4^	Institute of Physiology, Medical University of Innsbruck, 6020 Innsbruck, Austria

^5^	Department of Chemistry and Biochemistry, University of Notre Dame, Notre Dame, Indiana 46556, United States

*Corresponding authors:*

Clemens Decristoforo, Department of Nuclear Medicine, Anichstrasse 35, A-6020 Innsbruck, Austria, Tel: +4351250480951, email: [Clemens.Decristoforo@i-med.ac.at](mailto:Clemens.Decristoforo@i-med.ac.at)

**Table of content**

Instrumentation	4

Analytical [radio]-RP-HPLC	4

Preparative RP-HPLC	4

ESI-MS	5

^1^H-NMR	5

Synthesis of the labelling precursors	5

Materials	5

Synthesis of the FAPi alkyne derivative	6

Esterification: Methyl 6-methoxyquinoline-4-carboxylate	6

Demethylation: Methyl 6-hydroxyquinoline-4-carboxylate	8

Etherification: Methyl 6-(3-chloropropoxy)quinoline-4-carboxylate	9

Nucleophilic substitution & hydrolysis: 6-(3-(1-piperazinyl-4-prop-2-ynyl)propoxy)quinoline-4-carboxylic acid	11

Amide coupling: (S)-*N*-(2-(2-Cyano-4,4-difluoropyrrolidin-1-yl)-2-oxoethyl)-6-(3-(1-piperazinyl-4-prop-2- ynyl)propoxy)quinoline-4-carboxamide	13

Extraction of Fusarinine C (FSC)	15

[Fe]FSC derivatisation_Fmoc-*N*-amido-PEG2-[Fe]FSC	16

[Fe]FSC derivatization: NH_2_-PEG2-[Fe]FSC(PEG4-azide)2	17

[Fe]FSC derivatisation_NH_2_-PEG2-[Fe]FSC(PEG4-FAPi)2	18

ZW800-FFAPi synthesis	19

s775z-FFAPi synthesis	20

IRDye-FFAPi synthesis	21

SCy7-FFAPi synthesis	22

Ac-FFAPi synthesis	23

Radiolabelling and quality control by *radio*-HPLC and *radio*-iTLC	24

Distribution coefficient (LogD_pH7.4_), stability in human serum and protein binding	28

Lipophilicity determination	28

Human serum stability study for [^68^Ga]Ga-s775z-FFAPi	29

Competitive binding assays	29

Animal experiments	29

Metabolic *in vivo* stability	29

Metabolic stability for [^68^Ga]Ga-s775z-FFAPi	30

Metabolic stability for [^68^Ga]Ga-IRDye-FFAPi	30

*Ex vivo* biodistribution results based on radioactive signal analysis	30

*Ex vivo* biodistribution results based on fluorescence signal analysis	33

Optical properties	34

Statistical and data analysis	35

# Instrumentation

## Analytical [radio]-RP-HPLC

RP-HPLC analysis was performed on UltiMate 3000 system equipped with pump, autosampler, column compartment, diode array detector (Thermo Fisher Scientific, Vienna, Austria) and radio detector (GabiStar, Raytest; Straubenhardt, Germany).

Method A(A): A Jupiter 4 μm Proteo 90 Å 250 x 4.6 mm (Phenomenex Ltd. Aschaffenburg, Germany) column with flow rate of 1 mL/min and UV detection at 220 nm was used. Acetonitrile (ACN)/H_2_O + 0.1% trifluoroacetic acid (TFA) was used as mobile phase with the following multistep gradient: 0.0-3.0 min 10% ACN, 3.0-20.0 min 10-28.5% ACN, 20.0-20.1 min 28.5-70% ACN, 20.1-24.0 min 70% ACN, 24.0-24.1 min 70-10% ACN, 24.1-29.0 min 10% ACN.

Method A(B): A Jupiter 4 μm Proteo 90 Å 250 x 4.6 mm (Phenomenex Ltd. Aschaffenburg, Germany) column with flow rate of 1 mL/min and UV detection at 220 nm was used. Acetonitrile (ACN)/H_2_O + 0.1% trifluoroacetic acid (TFA) was used as mobile phase with the following multistep gradient: 0.0-3.0 min 10% ACN, 3.0-16.0 min 10-60% ACN, 16.0-18.0 min 60% ACN, 18.0-18.1 min 60-10% ACN, 18.1-22.0 min 10% ACN.

Method A(C): A Jupiter 4 μm Proteo 90 Å 250 x 4.6 mm (Phenomenex Ltd. Aschaffenburg, Germany) column with flow rate of 1 mL/min and UV detection at 220 nm was used. Acetonitrile (ACN)/H_2_O + 0.1% trifluoroacetic acid (TFA) was used as mobile phase with the following multistep gradient: 0.0-3.0 min 10% ACN, 3.0-16.0 min 10-60% ACN, 16.0-19.0 min 60-70% ACN, 19.0-21.0 min 70% ACN, 21.0-21.1 min 70-10% ACN, 21.1-25.0 min 10% ACN.

Method A(D): A Jupiter 4 μm Proteo 90 Å 250 x 4.6 mm (Phenomenex Ltd. Aschaffenburg, Germany) column with flow rate of 1 mL/min and UV detection at 220 nm was used. Acetonitrile (ACN)/H_2_O + 0.1% trifluoroacetic acid (TFA) was used as mobile phase with the following multistep gradient: 0.0-3.0 min 22% ACN, 3.0-9.0 min 22-27% ACN, 9.0-16.5 min 27-29% ACN, 16.5-16.6 min 29-70% ACN, 16.6-18.0 min 70% ACN, 18.0-18.1 min 70-22% ACN, 18.1-22.0 min 22% ACN.

Method A(E): A Jupiter 4 μm Proteo 90 Å 250 x 4.6 mm (Phenomenex Ltd. Aschaffenburg, Germany) column with flow rate of 1 mL/min and UV detection at 220 nm was used. Acetonitrile (ACN)/H_2_O + 0.1% trifluoroacetic acid (TFA) was used as mobile phase with the following multistep gradient: 0.0-3.0 min 22% ACN, 3.0-9.0 min 22-27% ACN, 9.0-30.5 min 27-35% ACN, 30.5-31.0 min 35-70% ACN, 31.0-32.0 min 70% ACN, 32.0-32.1 min 70-22% ACN, 32.1-36.0 min 22% ACN.

## Preparative RP-HPLC

RP-HPLC purification was performed on a UltiMate 3000 pump with UltiMate 3000 UV/Vis detector (Thermo Fisher Scientific, Vienna, Austria).

Method P(A): a Nucleodur 5 μm C18 HTec 250 x 16 mm (Macherey-Nagel, Düren, Germany) column with flow rate of 7 mL/min and UV detection at 220 nm was used. Acetonitrile (ACN)/H_2_O + 0.1% trifluoroacetic acid (TFA) was used as mobile phase with the following multistep gradient: 0.0-5.0 min 10% ACN, 5.0-31.0 min 10-30% ACN, 31.0-31.5 min 70% ACN, 31.5-35.0 min 70% ACN, 35.0-35.5 min 10% ACN, 35.5-43.0 min 10% ACN.

Method P(B): a Nucleodur 7 μm C18 HTec 250 x 21 mm (Macherey-Nagel, Düren, Germany) column with flow rate of 15 mL/min and UV detection at 440 nm was used. Acetonitrile (ACN)/H_2_O + 0.1% trifluoroacetic acid (TFA) was used as mobile phase with the following multistep gradient: 0.0-5.0 min 3% ACN, 5.0-7.0 min 3-12% ACN, 7.0-17.0 min 12-22% ACN, 17.0-17.5 min 22-3% ACN, 17.5-27.0 min 3% ACN.

Method P(C): a Nucleodur 5 μm C18 HTec 250 x 16 mm (Macherey-Nagel, Düren, Germany) column with flow rate of 8 mL/min  and UV detection at 440 nm was used. Acetonitrile (ACN)/H_2_O + 0.1% trifluoroacetic acid (TFA) was used as mobile phase with the following multistep gradient: 0.0-5.0 min 10% ACN, 5.0-25.0 min 10-65% ACN, 25.0-25.1 min 65-10% ACN, 25.1-34.0 min 10% ACN.

Method P(D): a Nucleodur 5 μm C18 HTec 250 x 16 mm (Macherey-Nagel, Düren, Germany) column with flow rate of 8 mL/min  and UV detection at 440 nm was used. Acetonitrile (ACN)/H_2_O + 0.1% trifluoroacetic acid (TFA) was used as mobile phase with the following multistep gradient: 0.0-5.0 min 10% ACN, 5.0-11.0 min 10-26.5% ACN, 11.0-20.0 min 26.5-41.6% ACN, 20.0-20.1 min 41.6-10% ACN, 20.1-30.0 min 10% ACN.

Method P(E): a Nucleodur 5 μm C18 HTec 250 x 16 mm (Macherey-Nagel, Düren, Germany) column with flow rate of 8 mL/min  and UV detection at 660 nm was used. Acetonitrile (ACN)/H_2_O + 0.1% trifluoroacetic acid (TFA) was used as mobile phase with the following multistep gradient: 0.0-3.0 min 17% ACN, 3.0-26.0 min 17.0-35.0% ACN, 26.0-26.1 min 35.0-17.0% ACN, 26.1-30.0 min 17% ACN.

Method P(F): a Nucleodur 5 μm C18 HTec 250 x 16 mm (Macherey-Nagel, Düren, Germany) column with flow rate of 8 mL/min  and UV detection at 660 nm was used. Acetonitrile (ACN)/H_2_O + 0.1% trifluoroacetic acid (TFA) was used as mobile phase with the following multistep gradient: 0.0-3.0 min 17% ACN, 3.0-33.0 min 17.0-40.0% ACN, 33.0-33.1 min 40.0-17.0% ACN, 33.1-40.0 min 17% ACN.

## ESI-MS

All ESI-MS experiments were carried out with LCMS-2050 Nexera (Shimadzu, Kyoto, Japan) using the following conditions: scan range (m/z 200.0 – 2000; sampling 500 msec /2Hz; mobile phase 30/70 H2O/ACN +0.1 % Formic Acid; flow rate of 0.5 mL/min). Data were acquired and evaluated with LabSolutions software (Shimadzu, Kyoto, Japan).

## ^1^H-NMR

^1^H-NMR spectra were recorded on a Varian Gemini 200 spectrometer 199.98 MHz. The center of the solvent multiplet (DMSO-d_6_) was used as internal standard (chemical shifts in δ ppm), which was related to TMS with δ 2.49 ppm.

# Synthesis of the labelling precursors

## Materials

All commercially available chemicals, reagents and solvents were of analytical grade and were used without further purification. Only high-purity water (18 mΩ) was employed. Coupling reagents *O*-(7-azabenzotriazol-1-yl)-*N,N,N´,N´-*tetramethyluronium-hexafluorophosphate (HATU) and 1-hydroxy-7-azabenzotriazole (HOAt) were obtained from GenScript Biotech Corporation (Piscataway, NJ, USA). 6-Methoxyquinoline-4-carboxylic acid was purchased from TCI Deutschland GmbH (Eschborn, Germany) while (S)-4,4-difluoro-1-glycylpyrrolidine-2-carbonitrile 2,2,2-trifluoroacetate was obtained from Advanced ChemBlock Inc (Hayward, CA, USA). Fmoc-*N*-amido-PEG2 acid was obtained from Tebubio GmbH (Offenbach am Main, Germany). Sulfo-Cyanine7 carboxylic acid and tris(3-hydroxypropyltriazolylmethyl)amine were purchased from Lumiprobe GmbH (Hannover, Germany), while azido-PEG4-acid was obtained from BroadPharm (San Diego, CA, USA) and IRDye800CW NHS ester from LI-COR GmbH (Bad Homburg, Germany). ZW800-1 carboxylic acid was a kind gift by Prof Dr. John V. Frangioni (The Curadel Companies, Natick, MA, USA). s775z carboxylic acid was kindly provided by Prof. Bladley D. Smith (University of Notre Dame, Notre Dame, IN, USA). All other reagents were purchased from Sigma-Aldrich Handels GmbH (Vienna, Austria) and VWR International GmbH (Vienna, Austria).

## Synthesis of the FAPi alkyne derivative

**Fig. S1** Synthetic scheme of the FAPi alkyne derivative

## Esterification: Methyl 6-methoxyquinoline-4-carboxylate

After 6-methoxyquinoline-4-methyl carboxylic acid **1** (500 mg, 2.46 mmol) was suspended in MeOH (20 mL), and five drops of concentrated H_2_SO_4_ were added under ice cooling, the solution was stirred at 60 °C overnight.

The solvent was evaporated under reduced pressure and the residue was washed with saturated sodium bicarbonate solution before extracting three times with dichloromethane. The combined organic phases were washed one time with brine and dried over sodium sulfate. The solvent was evaporated ~~by rotovap~~ and the residue was dried under vacuum. The title compound was obtained as light yellow solid (442 mg, 2.04 mmol, 83%).

^1^H-NMR (400 MHz, DMSO-d_6_): δ (ppm) = 8.88 (d, J = 4.4 Hz, 1H, A), 8.07 (d, J = 2.8 Hz, 1H, E), 8.03 (d, J = 9.2 Hz, 1H, C), 7.93 (d, J = 4.4 Hz, 1H, B), 7.50 (dd, J = 9.2, 2.8 Hz, 1H, D), 3.98 (s, 3H, OCH_3_), 3.91 (s, 3H, OCH_3_).

**Fig. S2** ^1^H NMR spectrum of methyl 6-methoxyquinoline-4-carboxylate

## Demethylation: Methyl 6-hydroxyquinoline-4-carboxylate

Methyl 6-methoxyquinoline-4- carboxylate **2** (0.240 g, 1.11 mmol) was dissolved in 6 mL of dry dichloromethane under argon atmosphere. A solution of BBr_3 _(3 mL, 1M in DCM (1.5 mmol)) was added dropwise to the reaction under ice cooling. The reaction mixture was stirred overnight at RT.

Subsequently, the solvent was evaporated under argon flow. Saturated sodium bicarbonate solution was added and the solution was extracted with ethyl acetate three times. The combined organic phases were dried over sodium sulfate, solvent was removed under reduced pressure, and the residue was dried in vacuo. Compound **3** was obtained as yellow solid (93 mg, 0.46 mmol, 41 %).

^1^H-NMR (400 MHz, DMSO-d_6_): δ (ppm) = 10.33 (s, 1H, G), 8.79 (d, J = 4.4 Hz, 1H, A), 7.97-7.95 (signals overlapped, 2H, C, E), 7.85 (d, J = 4.4 Hz, 1H, B), 7.38 (dd, J = 9.0, 2.6 Hz, 1H, D), 3.95 (s, 3H, F).

**Fig. S3** ^1^H NMR spectrum of methyl 6-hydroxyquinoline-4-carboxylate

## Etherification: Methyl 6-(3-chloropropoxy)quinoline-4-carboxylate

Methyl 6-hydroxyquinoline-4-carboxylate **3** (134 mg, 0.66 mmol) and Cs_2_CO_3_ (1.07 g, 3.28 mmol) were dissolved in dry DMF (6 mL), and subsequently 1-chloro-3-iodopropane (470 mg, 2.30 mmol, 0.257 mL) was added. The reaction was stirred at 60°C overnight. In the following, the solvent was removed under reduced pressure. The residue was partitioned between diethyl ether and water and the aqueous phase was extracted with diethyl ether four times. The combined organic phases were washed with brine, dried over sodium sulfate and the solvent was dried. The crude product was purified by column chromatography (petroleum ether/ethyl acetate 2/1) using silica. The desired compound **4** was obtained as yellow oil (120 mg, 0.43 mmol, 65 %).

^1^H-NMR (400 MHz, DMSO-d_6_): δ (ppm) = 8.88 (d, J = 4.4 Hz, 1H, A), 8.09 (d, J = 2.4 Hz, 1H, E), 8.04 (d, J = 9.2 Hz, 1H, C), 7.93 (d, J = 4.4 Hz, 1 H, B), 7.52 (dd, J = 9.4, 2.8 Hz, 1H, D), 4.24 (t, J = 6.0 Hz, 2H, G), 3.97 (s, 3H, F), 3.85 (t, J = 6.4 Hz, 2H, I), 2.28-2.24 (m, 2H, H).

**Fig. S4** ^1^H NMR spectrum of methyl 6-(3-chloropropoxy)quinoline-4-carboxylate

## Nucleophilic substitution & hydrolysis: 6-(3-(1-piperazinyl-4-prop-2-ynyl)propoxy)quinoline-4-carboxylic acid

To a solution of methyl 6-(3-chloropropoxy)quinoline-4-carboxylate **4** (117 mg, 0.42 mmol ) and 1-prop-2-yn-1-yl)piperazine (156 mg,1.26 mmol) in DMF (5 mL) was added potassium iodide (416 mg, 2.50 mmol). The reaction mixture was stirred under argon atmosphere at 60 °C overnight. After the solvent was removed, saturated sodium bicarbonate solution was added and ethyl acetate was used to extract three times. The combined organic phases were dried over sodium sulfate and the solvent was evaporated under reduced pressure. After the crude product **5** was dissolved in a mixture of methanol and water (3 mL, 3/1), sodium hydroxide solution (2 M, 0.3 mL) was added, and the resulting mixture was stirred at RT for 120 min. Then, HCl was used to neutralize and the solvent was dried under vacuum to obtain the crude product **6** (132 mg).

^1^H-NMR (400 MHz, DMSO-d_6_): δ (ppm) = 8.63 (d, J = 4.4 Hz, 1H, A), 8.18 (d, J = 2.8 Hz, 1H, E), 7.84 (d, J = 9.2 Hz, 1H, C), 7.53 (d, J = 4 Hz, 1H, B), 7.31 (dd, J = 9.2, 2.8 Hz, 1H, D), 4.06 (t, J = 6.4 Hz, 2H, F), 3.33-3.30 (m, covered by the H_2_O signal, 8H, I), 3.22 (d, J = 2.4 Hz, partially covered by the H_2_O peak, 2H, L), 3.11 (t, J = 2.4 Hz, 2H, H), 2.49 (t, covered by the DMSO peak, 1H, M), 1.93 (m, 2H, G)

**Fig. S5** ^1^H NMR spectrum of 6-(3-(1-piperazinyl-4-prop-2-ynyl)propoxy)quinoline-4-carboxylic acid

## Amide coupling: (S)-*N*-(2-(2-Cyano-4,4-difluoropyrrolidin-1-yl)-2-oxoethyl)-6-(3-(1-piperazinyl-4-prop-2- ynyl)propoxy)quinoline-4-carboxamide

6-(3-(1-Piperazinyl-4-prop-2-ynyl)propoxy)quinoline-4-carboxylic acid **6** (estimated 198 mg, 0.56 mmol), HOAT (153 mg, 1.12 mmol) and HATU (427.6 mg, 1.12 mmol) were dissolved in dry DMF (2mL).

The resulting solution was alkalized using DIPEA (243 μL, 1.4 mmol) and incubated for 10 min. (S)-1-(2-aminoacetyl)-4,4-difluoropyrrolidine-2-carbonitrile (170 mg , 0.56 mmol) was dissolved separately in dry DMF (1 mL) and DIPEA was added (194 μL, 1.12 mmol). The two solutions were combined and the resulting mixture was stirred overnight at RT. In the following, the reaction mixture was quenched with water and the solvent was evaporated under reduced pressure. Purification *via* preparative RP-HPLC (gradient P(A); t_R_ = 20.3 min) yielded compound **7** (78.9 mg, 15 mmol, 35 %) with a purity > 99 % which was confirmed by analytical RP-HPLC (gradient A(A); t_R_ = 14.7 min).

ESI/MS: *m/z* [M+ H^+^] = 525.2 [C_27_H_30_F_2_N_6_O_3_^+^], exact mass (monoisotopic): 525.2 (calculated); *m/z* [M+ H^+^+ K^+^] = 283.8 [C_27_H_31_F_2_N_6_O_3_K^2+^], exact mass (monoisotopic): 282.1 (calculated).

**Fig. S6** UV-VIS HPLC chromatogram of FAPi alkyne

**Fig. S7** MS spectrum of FAPi alkyne

|  | **R** | **Yield / %** | **Precursor** |
| --- | --- | --- | --- |
| **5a** | ZW800 acid | 45.8 | ZW800-FFAPi |
| **5b** | s775z acid | 44.4 | s775z-FFAPi |
| **5c** | IRDye800CW NHS ester | 57.1 | IRDye-FFAPi |
| **5d** | SulfoCy7 acid | 46.2 | Scy7-FFAPi |
| **5e** | AcOH | 60.0 | Ac-FFAPi |

**Fig. S8** Synthetic scheme of the prepared ligands

Extraction of Fusarinine C (FSC) **(compound 1 in Fig. S1)**

The siderophore-based natural product fusarinine C (FSC) (**1**) was obtained from 5 L of Aspergillus fumigatus ∆sidG culture prepared under iron deficiency as described by Schrettl and co-workers [1]. After filtering the culture supernatant, FeSO_4_ or FeCl_3_ was added in excess to a final concentration of 10 mM obtaining a red coloured solution. The iron complexation step is necessary to prevent the involvement of the hydroxamates in any side reactions during the synthesis. 

The sediment present in the solution was separated by centrifugation (5 min at 6000xg rpm and 25°C).

After preparative RP-HPLC purification (gradient P(B); t_R_ = 14.2 min) and freeze-drying, 735 mg of a red-brown powder was obtained with a purity > 94 % confirmed by analytical RP-HPLC (gradient A(B); t_R_ = 9.2 min). MS: *m/z* [M+H^+^] = 780.17 [C_33_H_52_FeN_6_O_12_^+^]; exact mass: 780.30 (calculated)].

**Fig. S9** UV-VIS HPLC chromatogram of [Fe]FSC

**Fig. S10** MS spectrum of [Fe]FSC

[Fe]FSC derivatisation_Fmoc-*N*-amido-PEG2-[Fe]FSC **(compound 2 in Fig. S1)**

98.8 mg of Fmoc-PEG2-acid (247.4 µmol, 1.0 eq.), 188.1 mg of HATU (494.7 µmol, 2.0 eq.) and 67.3 mg of HOAt (494.7 µmol, 2.0 eq.) were dissolved in 9.4 mL of dry DMF. The pH was adjusted to 9 with 215 µL of DIPEA and the resulting solution was allowed to rest for approximately 10 minutes.

674.9 mg of [Fe]FSC (**1**) (865.7 µmol, 3.5 eq.) were dissolved in 23 mL of dry DMF, then 250 µL of DIPEA were added. The two solutions were mixed and the pH checked and adjusted to 9 if necessary.

After 1 h, the solution was diluted 1:1 with water and then dried by rotary evaporation and the residue dissolved in a few millilitres of H_2_O/ACN 1/1.

Preparative RP-HPLC purification (gradient P(C); t_R_ = 20.3 min) yielded 133.9 mg (115.5 µmol, 46.7%) of Fmoc-N-amido-PEG2-[Fe]FSC (**2**) with a purity > 95 % confirmed by analytical RP-HPLC (gradient A(B); t_R_ = 14.6 min). ESI/MS: *m/z* [M+ H^+^] = 1161.1 [C_55_H_75_FeN_7_O_17_^+^], exact mass (monoisotopic): 1161.5 (calculated); *m/z* [M+2 H^+^] = 581.4 [C_55_H_76_FeN_7_O_17_^2+^], exact mass (monoisotopic): 581.2 (calculated).

**Fig. S11** UV-VIS HPLC chromatogram of Fmoc-*N*-amido-PEG2-[Fe]FSC

**Fig. S12** MS spectrum of Fmoc-*N*-amido-PEG2-[Fe]FSC

[Fe]FSC derivatization: NH_2_-PEG2-[Fe]FSC(PEG4-azide)2  **(compound 3 in Fig. S1)**

132 mg of Fmoc-*N*-amido-PEG2-[Fe]FSC (**2**) (113.7 µmol, 1.0 eq.) were dissolved in 4.4 mL of dry DMF and the pH adjusted to 9 with DIPEA.

216.1 mg of HATU (568.5 µmol, 2.0 eq.), 77.4 mg of HOAt (568.5 µmol, 2.0 eq.), were dissolved with 2.7 mL of dry DMF and then mixed with 82.8 mg of azido-PEG4-acid (284.2 µmol, 2.5 eq.). The pH was corrected to 9 with 300 µL of DIPEA and the resulting solution was allowed to rest for approximately 10 min. Thereafter it was added dropwise to the solution of the siderophore derivative, the final pH checked and adjusted to 9. After 1h the reaction was monitored by analytical RP-HPLC.

For the deprotection of the Fmoc group 308 µL of piperidine were added to the crude solution in order to reach a 4% (v/v) concentration and the resulting solution was stirred for 1h. After this time, the solution was diluted 1:1 with H_2_O, neutralized with HCl 1M and then dried by rotary evaporation. The residue was dissolved in a few millilitres of H_2_O + 25% (v/v) ACN.

Preparative RP-HPLC purification (gradient P(C); t_R_ = 20.2 min) yielded 111 mg (74.7 µmol, 65.7%) of NH_2_-PEG2-[Fe]FSC(PEG4-azide)_2 _(**3**) with a purity > 90 % confirmed by analytical RP-HPLC (gradient A(C); t_R_ = 14.4 min). ESI/MS: *m/z* [M+ H^+^] = 1484.7 [C_62_H_103_FeN_13_O_25_^+^], exact mass (monoisotopic): 1485.7 (calculated); *m/z* [M+2 H^+^] = 743.5 [C_62_H_104_FeN_13_O_25_^2+^], exact mass (monoisotopic): 743.4 (calculated).

**Fig. S13** UV-VIS HPLC chromatogram of NH_2_-PEG2-[Fe]FSC(PEG4-azide)_2_

**Fig. S14** MS spectrum of NH_2_-PEG2-[Fe]FSC(PEG4-azide)_2_

 

[Fe]FSC derivatisation_NH_2_-PEG2-[Fe]FSC(PEG4-FAPi)2  **(compound 4 in Fig. S1)**

45 mg of NH_2_-PEG2-[Fe]FSC(PEG4-azide)_2_ (**3**) (30.3 µmol, 1.0 eq.) and 39.7 mg of FAPi alkyne (75.7 µmol, 2.5 eq.) were dissolved together in 4 mL of H_2_O/tBuOH 1/1 and degassed with Argon flow for 10 min. 131.6 mg of THPTA (302.9 µmol, 10.0 eq) and 95.3 mg of Cu(ACN)_4_ catalyst (302.9 µmol, 10.0 eq) were dissolved separately in 1 mL of DMF and degassed with Argon flow for 15 min, then mixed together to give a clear green solution and degassed for further 5 min. Eventually the catalyst solution was added to the solution of azide and alkyne and let react under Argon atmosphere for 1 hour.

The crude mixture was concentrated by rotary evaporation, the residue dissolved in 1 mL of H_2_O + 30% (v/v) ACN and purified by preparative RP-HPLC (gradient P(D); t_R_ = 16.6 min) yielding 60.3 mg (23.8 µmol, 78.5%) of NH_2_-PEG2-[Fe]FSC(PEG4-FAPi)_2 _(**4**) with a purity > 88 % confirmed by analytical RP-HPLC (gradient A(C); t_R_ = 11.6 min). ESI/MS: *m/z* [M+2 H^+^] = 1267.7 [C_116_H_164_F_4_FeN_25_O_31_^2+^], exact mass (monoisotopic): 1267.6 (calculated); *m/z* [M+3 H^+^] = 845.7 [C_116_H_165_F_4_FeN_25_O_31_^3+^], exact mass (monoisotopic): 845.4 (calculated).

**Fig. S15** UV-VIS HPLC chromatogram of NH_2_-PEG2-[Fe]FSC(PEG4-FAPi)_2_

**Fig. S16** MS spectrum of NH_2_-PEG2-[Fe]FSC(PEG4-FAPi)_2_

ZW800-FFAPi synthesis **(compound 5a in Fig. S1)**

7.8 mg of NH_2_-PEG2-[Fe]FSC(PEG4-FAPi)_2_ (**4**) (3.1 µmol, 1.3 eq.) were dissolved in 390 µL of dry DMF and the pH adjusted to 9 with DIPEA.

3.6 mg of HATU (9.5 µmol, 4.0 eq.) and 1.3 mg of HOAt (9.5 µmol, 4.0 eq.) were dissolved in 100 µL of dry DMF and then mixed with 2.5 mg of ZW800-1 carboxylic acid (2.4 µmol, 1.0 eq.). The pH was corrected to 9 with 0.5 µL of DIPEA and the resulting solution was allowed to rest for approximately 10 min. Subsequently, this mixture was added dropwise to the solution of the siderophore derivative and the resulting pH was adjusted to 9. After stirring 1h at RT the reaction was monitored by analytical RP-HPLC. 

For demetallation, the crude solution was initially diluted in a 1:1 ratio with H_2_O. Thereafter, an aqueous solution of EDTA (500 mM) was added to provide an excess of approximately 45 equivalents of EDTA over the siderophore derivative. The pH was adjusted to 4.5, and the solution was stirred at RT for 4 hours. Subsequently, the EDTA precipitate was removed by centrifugation and the solution was dried by rotary evaporation. The resulting crude residue was then dissolved in 1 mL of H_2_O/ACN 1/1 and purified by preparative RP-HPLC purification (gradient P(E); t_R_ = 20.7 min) yielding 3.8 mg (1.1 µmol, 45.8%) of ZW800-FFAPi (**5a**) with a purity > 96 % confirmed by analytical RP-HPLC (gradient A(D); t_R_ = 12.9 min). ESI/MS: *m/z* [M^3+^] = 1136.2 [C_167_H_232_F_4_N_29_O_39_S_2_^3+^], exact mass (monoisotopic): 1135.9 (calculated); *m/z* [M+H^4+^] = 852.6 [C_167_H_233_F_4_N_29_O_39_S_2_^4+^], exact mass (monoisotopic): 852.2 (calculated); *m/z* [M+2H^5+^] = 682.3 [C_167_H_234_F_4_N_29_O_39_S_2_^5+^], exact mass (monoisotopic): 681.9 (calculated).

**Fig. S17** UV-VIS HPLC chromatogram of ZW800-FFAPi

**Fig. S18** MS spectrum of ZW800-FFAPi

s775z-FFAPi synthesis **(compound 5b in Fig. S1)**

5.9 mg of NH_2_-PEG2-[Fe]FSC(PEG4-FAPi)_2_ (**4**) (2.4 µmol, 1.3 eq.) were dissolved in 300 µL of dry DMF and the pH was adjusted to 9 with DIPEA.

2.1 mg of HATU (5.5 µmol, 3.0 eq.) and 0.7 mg of HOAt (5.5 µmol, 3.0 eq.) were dissolved in 60 µL of dry DMSO and then mixed with 2.5 mg of s775z carboxylic acid (1.8 µmol, 1.0 eq.). The pH was corrected to 9 with DIPEA and the resulting solution was allowed to rest for approximately 10 min. Subsequently, this mixture was added dropwise to the solution of the siderophore derivative and the resulting pH was adjusted to 9. After stirring 1h at RT the reaction was monitored by analytical RP-HPLC. 

For demetallation, the crude solution was initially diluted in a 1:1 ratio with H_2_O. Thereafter, an aqueous solution of EDTA (500 mM) was added to provide an excess of approximately 55 equivalents of EDTA over the siderophore derivative. The pH was adjusted to 4.5, and the solution was stirred at RT for 4 hours. Subsequently, the EDTA precipitate was removed by centrifugation and the solution was dried by rotary evaporation. The resulting crude residue was then dissolved in 1 mL of H_2_O + 20% (v/v) ACN and purified by preparative RP-HPLC (gradient P(E); t_R_ = 19.6 min) yielding 3.1 mg (0.8 µmol, 44.4%) of s775z-FFAPi (**5b**) with a purity > 96 % confirmed by analytical RP-HPLC (gradient A(D); t_R_ = 12.0 min). ESI/MS: *m/z* [M^3+^] = 1279.4 [C_184_H_262_F_4_N_35_O_46_S_2_^3+^], exact mass (monoisotopic): 1279.3 (calculated); *m/z* [M+H^4+^] = 960.0 [C_184_H_263_F_4_N_35_O_46_S_2_^4+^], exact mass (monoisotopic): 959.7 (calculated).

**Fig. S19** UV-VIS HPLC chromatogram of s775z-FFAPi

**Fig. S20** MS spectrum of s775z-FFAPi

IRDye-FFAPi synthesis **(compound 5c in Fig. S1)**

7.1 mg of NH_2_-PEG2-[Fe]FSC(PEG4-FAPi)_2_ (**4**) (2.8 µmol, 1.3 eq.) were dissolved in 355 µL of dry DMF and then mixed with 2.5 mg of IRDye 800CW NHS ester (2.1 µmol, 1.0 eq.). The pH was corrected to 9 with DIPEA and the resulting solution was stirred 1h at RT before being monitored by analytical RP-HPLC. 

For demetallation, the crude solution was initially diluted in a 1:1 ratio with H_2_O. Thereafter, an aqueous solution of EDTA (500 mM) was added to provide an excess of approximately 50 equivalents of EDTA over the siderophore derivative. The pH was adjusted to 4.5, and the solution was stirred at RT for 4 hours. Subsequently, the EDTA precipitate was removed by centrifugation and the solution was dried by rotary evaporation. The resulting crude residue was then dissolved in 1 mL of H_2_O + 20% (v/v) ACN and purified by preparative RP-HPLC (gradient P(E); t_R_ = 25.5 min) yielding 4.3 mg (1.2 µmol, 57.1%) of IRDye-FFAPi (**5c**) with a purity > 98 % confirmed by analytical RP-HPLC (gradient A(D); t_R_ = 16.0 min). ESI/MS: *m/z* [M+2H^3+^] = 1155.8 [C_162_H_220_F_4_N_27_O_45_S_4_^3+^], exact mass (monoisotopic): 1155.8 (calculated); *m/z* [M+3H^4+^] = 867.3 [C_162_H_221_F_4_N_27_O_45_S_4_^4+^], exact mass (monoisotopic): 867.1 (calculated).

**Fig. S21** UV-VIS HPLC chromatogram of IRDye-FFAPi

**Fig. S22** MS spectrum of IRDye-FFAPi

SCy7-FFAPi synthesis **(compound 5d in Fig. S1)**

4.2 mg of NH_2_-PEG2-[Fe]FSC(PEG4-FAPi)_2_ (**4**) (1.7 µmol, 1.3 eq.) were dissolved in 210 µL of dry DMF and the pH adjusted to 9 with DIPEA.

1.5 mg of HATU (3.8 µmol, 3.0 eq.) and 0.5 mg of HOAt (3.8 µmol, 3.0 eq.) were dissolved in 75 µL of dry DMF and then mixed with 0.9 mg of SulfoCy7 carboxylic acid (1.3 µmol, 1.0 eq.). The pH was corrected to 9 with DIPEA and the resulting solution was allowed to rest for approximately 10 min. Subsequently, this mixture was added dropwise to the solution of the siderophore derivative and the resulting pH was adjusted to 9. After stirring 1h at RT the reaction was monitored by analytical RP-HPLC. 

For demetallation, the crude solution was initially diluted in a 1:1 ratio with H_2_O. Thereafter, an aqueous solution of EDTA (500 mM) was added to provide an excess of approximately 50 equivalents of EDTA over the siderophore derivative. The pH was adjusted to 4.5, and the solution was stirred at RT for 4 hours. Subsequently, the EDTA precipitate was removed by centrifugation and the solution was dried by rotary evaporation. The resulting crude residue was then dissolved in 1 mL of H_2_O + 20% (v/v) ACN and purified by preparative RP-HPLC (gradient P(F); t_R_ = 28.1 min) yielding 1.9 mg (0.6 µmol, 46.2%) of SCy7-FFAPi (**5d**) with a purity > 98 % confirmed by analytical RP-HPLC (gradient A(E); t_R_ = 21.8 min). ESI/MS: *m/z* [M+2H^3+^] = 1058.4 [C_153_H_210_F_4_N_27_O_38_S_2_^3+^], exact mass (monoisotopic): 1057.8 (calculated); *m/z* [M+3H^4+^] = 793.9 [C_153_H_211_F_4_N_27_O_38_S_2_^4+^], exact mass (monoisotopic): 793.6 (calculated).

**Fig. S23** UV-VIS HPLC chromatogram of SCy7-FFAPi

**Fig. S24** MS spectrum of SCy7-FFAPi

Ac-FFAPi synthesis **(compound 5e in Fig. S1)**

5.1 mg of NH_2_-PEG2-[Fe]FSC(PEG4-FAPi)_2_ (**4**) (2.0 µmol, 0.4 eq.) were dissolved in 255 µL of dry DMF and the pH adjusted to 9 with DIPEA.

5.7 mg of HATU (15.0 µmol, 3.0 eq.) and 2.0 mg of HOAt (15.0 µmol, 3.0 eq.) were dissolved in 158 µL of dry DMF and then mixed with 0.3 mg of glacial acetic acid (5.0 µmol, 1.0 eq.). The pH was corrected to 9 with DIPEA and the resulting solution was allowed to rest for approximately 10 min. Subsequently, this mixture was added dropwise to the solution of the siderophore derivative and the resulting pH was adjusted to 9. After stirring 1h at RT the reaction was monitored by analytical RP-HPLC. 

For demetallation, the crude solution was initially diluted in a 1:1 ratio with H_2_O. Thereafter, an aqueous solution of EDTA (500 mM) was added to provide an excess of approximately 50 equivalents of EDTA over the siderophore derivative. The pH was adjusted to 4.5, and the solution was stirred at RT for 4 hours. Subsequently, the EDTA precipitate was removed by centrifugation and the solution was dried by rotary evaporation. The resulting crude residue was then dissolved in 1 mL of H_2_O + 20% (v/v) ACN and purified by preparative RP-HPLC (gradient P(E); t_R_ = 20.4 min) yielding 2.9 mg (1.2 µmol, 60.0%) of Ac-FFAPi (**5e**) with a purity > 98 % confirmed by analytical RP-HPLC (gradient A(D); t_R_ = 12.9 min). ESI/MS: *m/z* [M+2H^2+^] = 1261.9 [C_118_H_169_F_4_N_25_O_32_^2+^], exact mass (monoisotopic): 1262.1 (calculated); *m/z* [M+3H^3+^] = 841.9 [C_118_H_170_F_4_N_25_O_32_^3+^], exact mass (monoisotopic): 841.7 (calculated).

**Fig. S25** UV-VIS HPLC chromatogram of Ac-FFAPi

**Fig. S26** MS spectrum of Ac-FFAPi

# Radiolabelling and quality control by *radio*-HPLC and *radio*-iTLC

^68^GaCl_3_ was obtained from a commercial ^68^Ge/^68^Ga generator (Eckert and Ziegler, Berlin, Germany) eluted with 0.1 N HCl solution (Rotem Industries, Dimona, Israel). The fractionated elution method was used in order to increase the ratio of activity to volume to its maximum (150-200 MBq in 1.5 mL). 

For Gallium-68 labelling, 5 nmol of the precursors were mixed with 200 µL eluate (30-40 MBq) and with 42 µL of 1.1 M sodium acetate solution to get a final pH of 4.5. The resulting solution was then agitated at RT for 10 min. For Gallium-67 labelling, 5 nmol of the precursors were added to 200 µL of [^67^Ga]Ga Citrate solution (Curium Netherlands BV, Petten, The Netherlands) (10-75 MBq). The labelling process was completed in 10 minutes at 80°C under mild agitation.

Zirconium-89 labelling required neutralizing 7 uL (7 MBq) of zirconium oxalic solution (1 M, Revvity, Whaltham, US) using 6.7 µL of 1 M Na_2_CO_3_. After 3 min, 100 uL of 0.5 M HEPES buffer (pH 7) were added together with 7.5 nmol of precursor. The mixture was incubated for 30 min at 40°C under gentle shaking.

The radiochemical yield (RCY) was determined by radio instant thin layer chromatography (*radio*-ITLC) performed using iTLC-SG stripes (Agilent Technologies, Folsom, CA, USA). 0.1 M sodium citrate solution (pH 5) was used as eluent for Gallium-67/68 labelled derivatives (**Fig. S28** and **S30B**). The strips spotted with samples were analysed using a TLC scanner (Scan-RAM, LabLogistic, Sheffield, UK) or the Cyclone Plus (Perkin Elmer, Waltham, US). For Zirconium-89 labelled samples, the spotted iTLC-SG stripes were eluted using 0.05 M EDTA solution (pH 7) and then analysed with the Cyclone Plus (**Fig. S29B**).

Regardless of the elution system used, the labelled derivatives remained at the origin (Rf < 0.1), while the unbound radionuclide migrated to the solvent front (Rf > 0.9).

In addition, the purity of the Gallium-68 and Zirconium-89 radiolabelled compounds was determined by radio-RP-HPLC on a UltiMate 3000 system (Thermo Fisher Scientific, Vienna, Austria) equipped with an in-line radio detector (GabiStar, Raytest; Straubenhardt, Germany) (**Fig. S27** and **S29A**). A phenylbutyl bonded phase column (5 µm, 120 Å, 4.6×250 mm; YMC-Triart Phenyl, YMC, Kyoto, Japan) was used at a flow rate of 1 ml/min. Acetonitrile (ACN)/H_2_O + 0.1% trifluoroacetic acid (TFA) was used as mobile phase with the following multistep gradient: 0.0-3.0 min 10% ACN, 3.0-16.0 min 10-60% ACN, 16.0-18.0 min 60% ACN, 18.0-18.1 min 60-10% ACN, 18.1-22.0 min 10% ACN. 

For the analysis of the purity of the Gallium-67 radiolabelled compound (**Fig. S30A**), the following multistep gradient was employed: 0.0-3.0 min 0% ACN, 3.0-6.0 min 0-50% ACN, 6.0-10.0 min 50% ACN, 10.0-10.1 min 50-80% ACN, 10.1-13.0 min 80% ACN, 13.0-13.1 min 80-0% ACN, 13.1-15.0 min 0% ACN.

**Fig. S27** *Radio*-RP-HPLC chromatogram of Gallium-68 labelled derivatives

| [^68^Ga]Ga-ZW800-FFAPi | [^68^Ga]Ga-s775z-FFAPi |
| --- | --- |
| [^68^Ga]Ga-IRDye-FFAPi | [^68^Ga]Ga-SCy7-FFAPi |
| [^68^Ga]Ga-Ac-FFAPi |  |

**Fig. S28** *Radio*-iTLC scan of Gallium-68 labelled derivatives

 **A**

 **B**

**Fig. S29** A: *Radio*-RP-HPLC chromatogram of [^89^Zr]Zr-s775z-FFAPi. B: *Radio*-iTLC scan of the strip spotted with the labelling solution and eluted with 0.05 M EDTA solution (pH 7)

 **A**

 **B**

**Fig. S30** A: *Radio*-RP-HPLC chromatogram of [^67^Ga]Ga-s775z-FFAPi. B: *Radio*-iTLC scan of [^67^Ga]Ga-s775z-FFAPi

# Distribution coefficient (LogD_pH7.4_), stability in human serum and protein binding

LogD_pH7.4_, stability and protein binding was determined as previously described [2]. For stability and protein binding studies, samples were diluted with human serum to a concentration of 1.0 µM (^68^Ga) and 10 µM (^89^Zr) was performed and data were obtained by averaging the results of two independent experiments.

## Lipophilicity determination

**Fig. S31** Graphical representation of the lipophilicity determination (LogD) of the Gallium-68 labelled compounds obtained with 6 technical replicates. The asterisks represent the level of significance determined by using the *p* value (*: 0.01 < *p* < 0.05; **: 0.001 < *p* < 0.01; ***: *p* < 0.001)

## Human serum stability study for [^68^Ga]Ga-s775z-FFAPi

**Fig. S32** Radio-HPLC chromatograms of [^68^Ga]Ga-s775z-FFAPi after 1,2 and 4h of incubation in human serum

# Tumor cell lines

The human fibrosarcoma cell line HT1080 transfected with the human FAP (HT1080hFAP) and the wild type version (HT1080) were provided by Prof. Dr. Uwe Haberkorn (University Hospital Heidelberg, Germany) All cell culture media and reagents were purchased from Gibco, Invitrogen (Thermo Fisher Scientific, Austria) or Sigma-Aldrich (Darmstadt, Germany). Dulbecco’s Modified Eagle’s medium (DMEM) supplemented with 10% (v/v) fetal bovine serum, FBS (10270, Invitrogen, Thermo Fisher Scientific, Waltham, Massachussetts, US) and with 1% (v/v) penicillin-streptomycin-glutamine, PSG (10378, Gibco, Thermo Fisher Scientific, Waltham, Massachussetts, US), solution was used for cell culture. The cells were grown at 37°C in a humidified atmosphere with 5% carbon dioxide and were passaged 3 times per week using 2.5% trypsin-EDTA solution. 

# Competitive binding assays

The FAP binding affinities of [^nat^Ga]Ga-IRDye-FFAPi, [^nat^Ga]Ga-s775z-FFAPi and of [^nat^Ga]Ga-FAPI-46, used as reference, were evaluated in competitive assays against [^177^Lu]Lu-FAPI-46 on HT1080hFAP cells. 3 nmol of FAPI-46 were radiolabelled with 22 µL of [^177^Lu]LuCl_3_ solution (40 MBq), 30 µL of 2 M sodium acetate/acetic acid buffer solution with pH 5 and 14 µL of water. The labelling mixture was heated at 95°C for 30 min; afterwards it was diluted with PBS/0.5% (w/v) Bovine Serum Albumin (BSA) to a concentration of 4 nM of radioligand. Binding assays were carried out in 96-well filter plates (Multi-ScreenHTS-FB, Merck Group, Darmstadt, Germany) pretreated twice with 150 µL of culturing medium before 200000 HT1080hFAP cells per well were added. Competitive assays were performed in quadruplicates using increasing concentrations of competitor (0.00025-1000 nM) and a constant amount of [^177^Lu]Lu-FAPI-46 (∼25000 cpm, 1 nM). After 1 h of incubation at 37°C, the medium was removed by vacuum filtration and the ﬁlters were rapidly rinsed two times with ice-cold PBS/0.5% (w/v) BSA solution, removed and counted in a γ-counter. Half-maximal inhibitory concentration (IC_50_) values were calculated following non linear regression with Origin software (MicroCal Origin 6.1, Northampton, MA, USA). The results are presented as mean values derived from two independent experiments.

|  |  | **[**^**nat**^**Ga]Ga-FAPI-46** | **[**^**nat**^**Ga]Ga-s775z-FFAPi** | **[**^**68**^**Ga]Ga-IRDye-FFAPi** |
| --- | --- | --- | --- | --- |
| **FAP-affinity** | **IC50 (nM)** **± SD** | 2.19 ± 0.68 | 3.86 ± 3.08 | 3.66 ± 0.78 |

**Table S1** FAP binding affinity of [^nat^Ga]Ga-FAPI-46, [^nat^Ga]Ga-s775z-FFAPi and [^68^Ga]Ga-IRDye-FFAPi assessed by determining the half-maximal inhibitory concentration.

# Animal experiments

## General

The studies were performed using 7-9-week-old female BALB/c mice (Charles River Laboratories, Sulzfeld, Germany) or 9-10 week-old athymic female BALB/c nude mice (Envigo, Horst, The Netherlands). Animals were housed in groups of five per cage in standard polysulfone cages under controlled environmental conditions (temperature: 22 ± 2°C; humidity: 50 ± 10%; 12 h light/dark cycle). Food and water were provided ad libitum. Environmental enrichment, including nesting materials and tunnels, was provided to promote natural behaviors. Retro-orbital tracer injection, small animal imaging and sacrification by cervical dislocation were carried out under 2% isoflurane anesthesia (FORANE, Abbott Laboratories, Abbott Park, IL, USA). A minimum of 3 animals per group was chosen in the biodistribution experiments to achieve a basic level of statistical significance.

## Metabolic *in vivo* stability

One healthy BALB/c mouse was injected with 3.0 nmol of [^68^Ga]Ga-s775z-FFAPi or [^68^Ga]Ga-IRDye-FFAPi (14-16 MBq). 15 min p.i. the animals were sacrificed and urine and blood samples were collected. Before radio-RP-HPLC analysis, the blood samples were centrifuged for 2 min at 14,000 rpm. 100 µL of the supernatant from each sample was diluted 1:1 with ACN, agitated and centrifuged again to separate the protein pellet. An aliquot of the supernatant was diluted 1:1 with water and analysed. For the HPLC analysis the urine samples were solely diluted 1:100 with water.

## Metabolic stability for [^68^Ga]Ga-s775z-FFAPi

**Fig. S33** Radio-HPLC chromatograms for [68Ga]Ga-s775z-FFAPi injection solution (3.0 nmol,14.7 MBq) in mouse serum and urine 15 min p.i.

## Metabolic stability for [^68^Ga]Ga-IRDye-FFAPi

**Fig. S34** Radio-HPLC chromatograms for [^68^Ga]Ga-IRDye-FFAPi inection solution (3.0 nmol,16.2 MBq) in mouse serum and urine 15 min p.i.

## *Ex vivo* biodistribution results based on radioactive signal analysis

|  | **1 h p.i.** |  |  |  |  |
| --- | --- | --- | --- | --- | --- |
|  | **[**^**68**^**Ga]Ga-Ac-FFAPi** | **[**^**68**^**Ga]Ga-SCy7-FFAPi** | **[**^**68**^**Ga]Ga-IRDye-FFAPi** | **[**^**68**^**Ga]Ga-ZW800-FFAPi** | **[**^**68**^**Ga]Ga-s775z-FFAPi** |
| Blood | 9.32 ± 0.24 | 6.11 ± 0.54 | 6.61 ± 0.48 | 7.60 ± 0.43 | 5.59 ± 0.48 |
| Spleen | 1.87 ± 0.12 | 16.90 ± 3.27 | 1.55 ± 0.10 | 2.30 ± 0.23 | 1.40 ± 0.09 |
| Pancreas | 7.21 ± 0.40 | 4.44 ± 0.23 | 4.04 ± 0.30 | 6.38 ± 0.22 | 4.17 ± 0.18 |
| Stomach | 1.98 ± 0.06 | 2.18 ± 0.45 | 1.53 ± 0.29 | 1.83 ± 0.03 | 1.43 ± 0.15 |
| Intestine | 2.42 ± 0.07 | 1.84 ± 0.20 | 1.88 ± 0.19 | 2.33 ± 0.01 | 1.88 ± 0.16 |
| Kidneys | 3.02 ± 0.09 | 12.08 ± 1.79 | 6.75 ± 0.64 | 5.07 ± 0.28 | 3.78 ± 0.32 |
| Liver | 2.45 ± 0.07 | 19.66 ± 2.91 | 2.24 ± 0.18 | 5.10 ± 0.16 | 2.20 ± 0.03 |
| Heart | 3.86 ± 0.32 | 2.69 ± 0.34 | 2.60 ± 0.25 | 3.15 ± 0.24 | 2.17 ± 0.20 |
| Lung | 4.52 ± 1.38 | 4.86 ± 0.72 | 4.70 ± 0.59 | 4.22 ± 0.50 | 2.74 ± 0.33 |
| Muscle | 3.77 ± 0.28 | 1.93 ± 0.29 | 2.64 ± 0.49 | 3.66 ± 0.68 | 3.25 ± 0.49 |
| Femur | 7.96 ± 1.94 | 4.43 ± 0.70 | 7.12 ± 1.11 | 8.10 ± 1.11 | 5.68 ± 0.54 |

**Table S2** Ex vivo biodistribution data obtained at 1 h p.i. in healthy BALB/C mice (n = 4) with [⁶⁸Ga]Ga-s775z-FFAPi and [⁶⁸Ga]Ga-Ac-FFAPi (amount injected: 0.10 nmol, 0.5 MBq). Data are expressed as a percentage of the injected dose per gram (%ID/g), mean value ± standard deviation

| **1 h p.i.** |  |
| --- | --- |
|  | **[**^**89**^**Zr]Zr-s775z-FFAPi** |
| Blood | 6.32 ± 1.32 |
| Spleen | 1.61 ± 0.36 |
| Pancreas | 2.82 ± 0.75 |
| Stomach | 0.96 ± 0.28 |
| Intestine | 1.12 ± 0.25 |
| Kidneys | 2.42 ± 0.61 |
| Liver | 2.05 ± 0.41 |
| Heart | 2.19 ± 0.42 |
| Lung | 2.52 ± 0.63 |
| Muscle | 1.40 ± 0.28 |
| Femur | 3.08 ± 0.36 |

**Table S3** Ex vivo biodistribution data obtained at 1 h p.i. in healthy BALB/C mice (n = 4) with [^89^Zr]Zr-s775z-FFAPi (amount injected: 0.10 nmol, 0.08 MBq). Data are expressed as a percentage of the injected dose per gram (%ID/g), mean value ± standard deviation

| **Table S4A** |  | **[**^**67**^**Ga]Ga-ZW800-FFAPi** |  |  |
| --- | --- | --- | --- | --- |
|  |  | **1 h p.i.** | **4 h p.i.** | **1 day p.i.** |
| Blood |  | 3.39 ± 0.39 | 2.89 ± 0.35 | 1.20 ± 0.14 |
| Spleen |  | 3.50 ± 1.25 | 2.65 ± 0.15 | 2.83 ± 0.32 |
| Pancreas |  | 9.81 ± 2.69 | 7.71 ± 0.31 | 4.74 ± 0.30 |
| Stomach |  | 2.53 ± 0.34 | 1.99 ± 0.12 | 1.23 ± 0.11 |
| Intestine |  | 2.40 ± 0.48 | 1.89 ± 0.24 | 1.55 ± 0.23 |
| Kidneys |  | 5.83 ± 0.35 | 4.84 ± 0.20 | 4.47 ± 0.22 |
| Liver |  | 8.23 ± 1.04 | 7.79 ± 0.41 | 8.19 ± 1.31 |
| Heart |  | 2.17 ± 0.16 | 2.13 ± 0.21 | 1.30 ± 0.06 |
| Lung |  | 5.12 ± 1.81 | 2.58 ± 0.20 | 1.31 ± 0.22 |
| Muscle |  | 4.07 ± 0.86 | 3.51 ± 0.64 | 2.12 ± 0.42 |
| Femur |  | 10.91 ± 2.92 | 7.59 ± 1.12 | 5.12 ± 0.59 |
| HT1080hFAP tumor |  | 5.54 ± 0.38 | 5.09 ± 0.84 | 4.51 ± 0.49 |
| HT1080 tumor |  | 2.76 ± 0.72 | 3.41 ± 1.21 | 2.22 ± 0.05 |
| HT1080hFAP/HT1080 ratio |  | 2.04 ± 0.67 | 2.01 ± 0.74 | 1.88 ± 0.12 |
| HT1080hFAP/muscle |  | 1.41 ± 0.32 | 1.31 ± 0.70 | 2.31 ± 0.63 |
| HT1080hFAP/liver |  | 0.68 ± 0.05 | 0.66 ± 0.17 | 0.56 ± 0.07 |
| HT1080hFAP/kidney |  | 1.46 ± 0.80 | 1.28 ± 0.18 | 1.12 ± 0.20 |
|  |  |  |  |  |
| **Table S4B** | **[**^**67**^**Ga]Ga-IRDye-FFAPi** |  |  |  |
|  |  | **1 h p.i.** | **4 h p.i.** | **1 day p.i.** |
| Blood |  | 2.25 ± 0.36 | 1.62 ± 0.25 | 0.74 ± 0.08 |
| Spleen |  | 1.97 ± 0.34 | 1.77 ± 0.43 | 2.27 ± 0.15 |
| Pancreas |  | 4.19 ± 0.82 | 2.83 ± 0.05 | 2.11 ± 0.45 |
| Stomach |  | 1.90 ± 0.29 | 0.85 ± 0.03 | 0.68 ± 0.13 |
| Intestine |  | 1.12 ± 0.10 | 1.16 ± 0.16 | 0.99 ± 0.15 |
| Kidneys |  | 9.99 ± 0.87 | 9.98 ± 1.12 | 13.20 ± 1.53 |
| Liver |  | 1.77 ± 0.23 | 1.70 ± 0.24 | 3.45 ± 0.42 |
| Heart |  | 1.81 ± 0.42 | 1.10 ± 0.05 | 0.87 ± 0.06 |
| Lung |  | 4.16 ± 1.37 | 2.18 ± 0.44 | 0.94 ± 0.11 |
| Muscle |  | 2.32 ± 0.32 | 1.98 ± 0.29 | 1.24 ± 0.03 |
| Femur |  | 6.67 ± 1.50 | 8.24 ± 0.86 | 4.00 ± 0.89 |
| HT1080hFAP tumor |  | 7.04 ± 0.91 | 7.98 ± 0.42 | 7.48 ± 0.79 |
| HT1080 tumor |  | 1.95 ± 0.39 | 2.63 ± 1.31 | 1.50 ± 0.26 |
| HT1080hFAP/HT1080 ratio |  | 4.17 ± 0.80 | 3.37 ± 1.62 | 4.75 ± 1.16 |
| HT1080hFAP/muscle |  | 3.05 ± 0.20 | 4.16 ± 1.10 | 6.05 ± 0.69 |
| HT1080hFAP/liver |  | 4.00 ± 0.42 | 4.84 ± 1.22 | 2.17 ± 0.05 |
| HT1080hFAP/kidney |  | 0.71 ± 0.14 | 0.81 ± 0.10 | 0.57 ± 0.06 |
|  |  |  |  |  |
| **Table S4C** |  | **[**^**67**^**Ga]Ga-s775z-FFAPi** |  |  |
|  |  | **1 h p.i.** | **4 h p.i.** | **1 day p.i.** |
| Blood |  | 1.97 ± 0.42 | 1.94 ± 0.31 | 0.92 ± 0.07 |
| Spleen |  | 1.34 ± 0.11 | 2.04 ± 0.38 | 2.23 ± 0.28 |
| Pancreas |  | 5.55 ± 0.43 | 5.60 ± 0.65 | 3.45 ± 0.32 |
| Stomach |  | 1.40 ± 0.29 | 1.26 ± 0.17 | 0.99 ± 0.10 |
| Intestine |  | 1.50 ± 0.52 | 1.33 ± 0.12 | 1.00 ± 0.18 |
| Kidneys |  | 4.47 ± 0.44 | 4.47 ± 0.25 | 3.87 ± 0.31 |
| Liver |  | 1.81 ± 0.15 | 2.29 ± 0.23 | 3.39 ± 0.75 |
| Heart |  | 1.23 ± 0.14 | 1.33 ± 0.08 | 0.97 ± 0.06 |
| Lung |  | 2.50 ± 0.41 | 2.25 ± 0.49 | 1.05 ± 0.20 |
| Muscle |  | 2.13 ± 0.40 | 2.82 ± 0.48 | 2.00 ± 0.08 |
| Femur |  | 7.09 ± 1.67 | 7.13 ± 0.81 | 5.90 ± 1.35 |
| HT1080hFAP tumor |  | 7.57 ± 1.06 | 8.01 ± 0.76 | 5.44 ± 0.23 |
| HT1080 tumor |  | 1.92 ± 0.07 | 2.65 ± 1.10 | 1.64 ± 0.13 |
| HT1080hFAP/HT1080 ratio |  | 4.34 ± 0.18 | 2.68 ± 0.48 | 3.42 ± 0.59 |
| HT1080hFAP/muscle |  | 3.76 ± 1.40 | 3.21 ± 0.29 | 2.72 ± 0.22 |
| HT1080hFAP/liver |  | 4.25 ± 1.09 | 3.28 ± 0.21 | 1.68 ± 0.45 |
| HT1080hFAP/kidney |  | 1.69 ± 0.09 | 1.87 ± 0.31 | 1.41 ± 0.14 |

**Table S4** Ex vivo biodistribution data obtained at 1h, 4h and 1 day p.i. in HT1080hFAP/ HT1080 xenografted BALB/C nude mice (n = 3) with [⁶^7^Ga]Ga-ZW800-FFAPi (Table S4A), [⁶^7^Ga]Ga-IRDye-FFAPi (Table S4B) and [⁶^7^Ga]Ga-s775z-FFAPi (Table S4C) (amount injected: 0.25 nmol, 0.4 MBq). Data are expressed as a percentage of the injected dose per gram (%ID/g), mean value ± standard deviation

## *Ex vivo* biodistribution results based on fluorescence signal analysis

**Fig. S35** Visible (left) and fluorescent images (right) of selected harvested organs from mice injected with Gallium-67 labelled tracers and sacrificed at 1 h and 1 day p.i. For this purpose, the in vivo MS FX PRO small-animal imaging system (Bruker Biospin Corporation,Woodbridge, CT, USA) was used and image analysis was performed with Bruker MI SE software v. 7.1.1.20220 (Bruker Biospin Corporation,Woodbridge, CT, USA). The same filter set (excitation = 720 nm and emission = 790 nm) and identical illumination settings were considered (acquisition time = 5 s, f-stop = 2.8, field of view = 100 mm, and binning = 4 × 4). The fluorescence emission was reported as photons/s/mm2. P: pancreas, H: heart, Lu: lung, K: kidneys, L: liver, M: muscle, T(+): HT1080hFAP tumor, T(-): HT1080 tumor

**Fig. S36** Tumor-to-organ fluorescence signal ratios. Ratios were calculated by analyzing the fluorescent images of harvested organs from xenografted mice injected with Gallium-67 labelled tracers during *ex vivo* biodistribution. Regions of interest (ROIs) were manually drawn, and mean fluorescence intensities were determined using Bruker MI SE software v.7.1.1.20220 (Bruker Biospin Corporation, Woodbridge, CT, USA)

# Optical properties 

| Compound | MW (Da) | λEx (nm) | λEm (nm) | Stokes shift |
| --- | --- | --- | --- | --- |
| SulfoCy7 | 709.9 | 750 | 773 | 23 |
| SCy7-FFAPi | 3173.6 | 757 | 781 | 24 |
| IRDye-800CW | 1101.3 | 775 | 792 | 17 |
| IRDye-FFAPi | 3467.9 | 781 | 799 | 18 |
| ZW800 | 946.2 | 768 | 786 | 18 |
| ZW800-FFAPi | 3409.9 | 773 | 793 | 20 |
| s775z | 1376.7 | 775 | 797 | 22 |
| s775z-FFAPi | 3840.4 | 780 | 803 | 23 |

**Table S5** Molecular weight and optical properties of the single fluorophore and the corresponding derivatives. Maximum excitation and emission wavelengths for the conjugates diluted in PBS solution (pH = 7.4) were determined experimentally (n = 2) by using a Tecan Spark multimode plate reader (Tecan, Männedorf, Switzerland; top reading, fluorescence emission scan). Analogous values related to the unconjugated dyes were obtained from the manufacturers.

# Statistical and data analysis

All the statistical analyses were performed using Microsoft Office 365 Excel software (Microsoft Corporation, Redmond, WA, USA). The significance of 2 mean values was calculated using an unpaired/independent two-tailed Student’s t-test. The level of significance was determined by using the P value (*: 0.01 < P < 0.05; **: 0.001 < P < 0.01; ***: P < 0.001).

# Discussion of *in vitro* results

The incorporation of two PEG4-FAPi units enhanced the hydrophilicity of the FSC scaffold, as evidenced by the more negative LogD_pH7.4 _value of [^68^Ga]Ga-Ac-FFAPi (−2.51 ± 0.01) compared to that of the triacetylated FSC, TAFC (LogD_pH7.4 _= -2.08 ± 0.02) [3]. Despite the larger size, the hydrophilic character was comparable to the dimer [^68^Ga]Ga-DOTAGA.Glu(FAPI)_2_ (LogD_pH7.4 _= -2.48 ± 0.05) and not drastically lower than that of [^68^Ga]Ga-(FAPI-04)_2_ (LogD_pH7.4 _= -3.05 ± 0.07) [4, 5]. The lipophilicity of the bimodal compounds decreased with the number of charged groups and the presence of the shielding PEGylated arms (**Table 1** and **Fig. S31**). While the incorporation of SulfoCy7 resulted in slight increased lipophilicity compared to the control, IRDye800CW and ZW800 had no significant impact on the LogD_pH7.4_, indicating that their introduction was well tolerated. Notably, the cellular uptake of the different candidates appeared to increase with their hydrophilic character (**Fig. 2**). It should, however, be noted that the actual ratio between internalized and membrane-bound fractions may differ from the obtained values, as the glycine buffer used to quantify the membrane-bound fraction may not fully dissociate FAPI ligands from FAP, as previously reported [6]. 

No conclusive correlation was identified between lipophilicity, protein affinity and cellular uptake across the probes, suggesting that multiple fluorophore-related factors-such as size, charge distribution, polarity and flexibility-may influence these properties to varying degrees.

**References**

1.	Schrettl M, Bignell E, Kragl C, Sabiha Y, Loss O, Eisendle M, et al. Distinct roles for intra-and extracellular siderophores during Aspergillus fumigatus infection. PLoS pathogens. 2007;3:e128.

2.	Gariglio G, Bendova K, Hermann M, Olafsdottir A, Sosabowski JK, Petrik M, et al. Comparison of Two Chelator Scaffolds as Basis for Cholecystokinin-2 Receptor Targeting Bimodal Imaging Probes. Pharmaceuticals. 2024;17:1569.

3.	Kaeopookum P, Summer D, Pfister J, Orasch T, Lechner BE, Petrik M, et al. Modifying the siderophore triacetylfusarinine C for molecular imaging of fungal infection. Molecular imaging and biology. 2019;21:1097-106.

4.	Martin M, Ballal S, Yadav MP, Bal C, Van Rymenant Y, De Loose J, et al. Novel generation of FAP inhibitor-based homodimers for improved application in radiotheranostics. Cancers. 2023;15:1889.

5.	Zhong X, Guo J, Han X, Wu W, Yang R, Zhang J, et al. Synthesis and preclinical evaluation of a novel FAPI-04 dimer for cancer theranostics. Molecular Pharmaceutics. 2023;20:2402-14.

6.	van der Heide CD, Ma H, Hoorens MW, Campeiro JD, Stuurman DC, de Ridder CM, et al. In vitro and in vivo analyses of eFAP: a novel FAP-targeting small molecule for radionuclide theranostics and other oncological interventions. EJNMMI Radiopharmacy and Chemistry. 2024;9:55.
